# On Efficient and Scalable Computation of the Nonparametric Maximum Likelihood Estimator in Mixture Models

**Published:** 2024

**Authors:** Yangjing Zhang, Ying Cui, Bodhisattva Sen, Kim-Chuan Toh

**Affiliations:** Institute of Applied Mathematics, Academy of Mathematics and Systems Science, Chinese Academy of Sciences; Department of Industrial Engineering and Operations Research, University of California, Berkeley; Department of Statistics, Columbia University; Department of Mathematics, Institute of Operations Research and Analytics, National University of Singapore

**Keywords:** Augmented Lagrangian method, denoising, empirical Bayes, Gaussian location mixture model, heteroscedastic errors, semismooth Newton method, sparse second-order information

## Abstract

In this paper, we focus on the computation of the nonparametric maximum likelihood estimator (NPMLE) in multivariate mixture models. Our approach discretizes this infinite dimensional convex optimization problem by setting fixed support points for the NPMLE and optimizing over the mixing proportions. We propose an efficient and scalable semismooth Newton based augmented Lagrangian method (ALM). Our algorithm outperforms the state-of-the-art methods (Kim et al., 2020; Koenker and Gu, 2017), capable of handling $n\approx 10^{6}$ data points with $m\approx 10^{4}$ support points. A key advantage of our approach is its strategic utilization of the solution’s sparsity, leading to structured sparsity in Hessian computations. As a result, our algorithm demonstrates better scaling in terms of m when compared to the mixsqp method (Kim et al., 2020). The computed NPMLE can be directly applied to denoising the observations in the framework of empirical Bayes. We propose new denoising estimands in this context along with their consistent estimates. Extensive numerical experiments are conducted to illustrate the efficiency of our ALM. In particular, we employ our method to analyze two astronomy data sets: (i) Gaia-TGAS Catalog (Anderson et al., 2018) containing approximately 1.4 × 10^6^ data points in two dimensions, and (ii) a data set from the APOGEE survey (Majewski et al., 2017) with approximately 2.7 × 10^4^ data points.

## 1. Introduction

**Figure 1:**
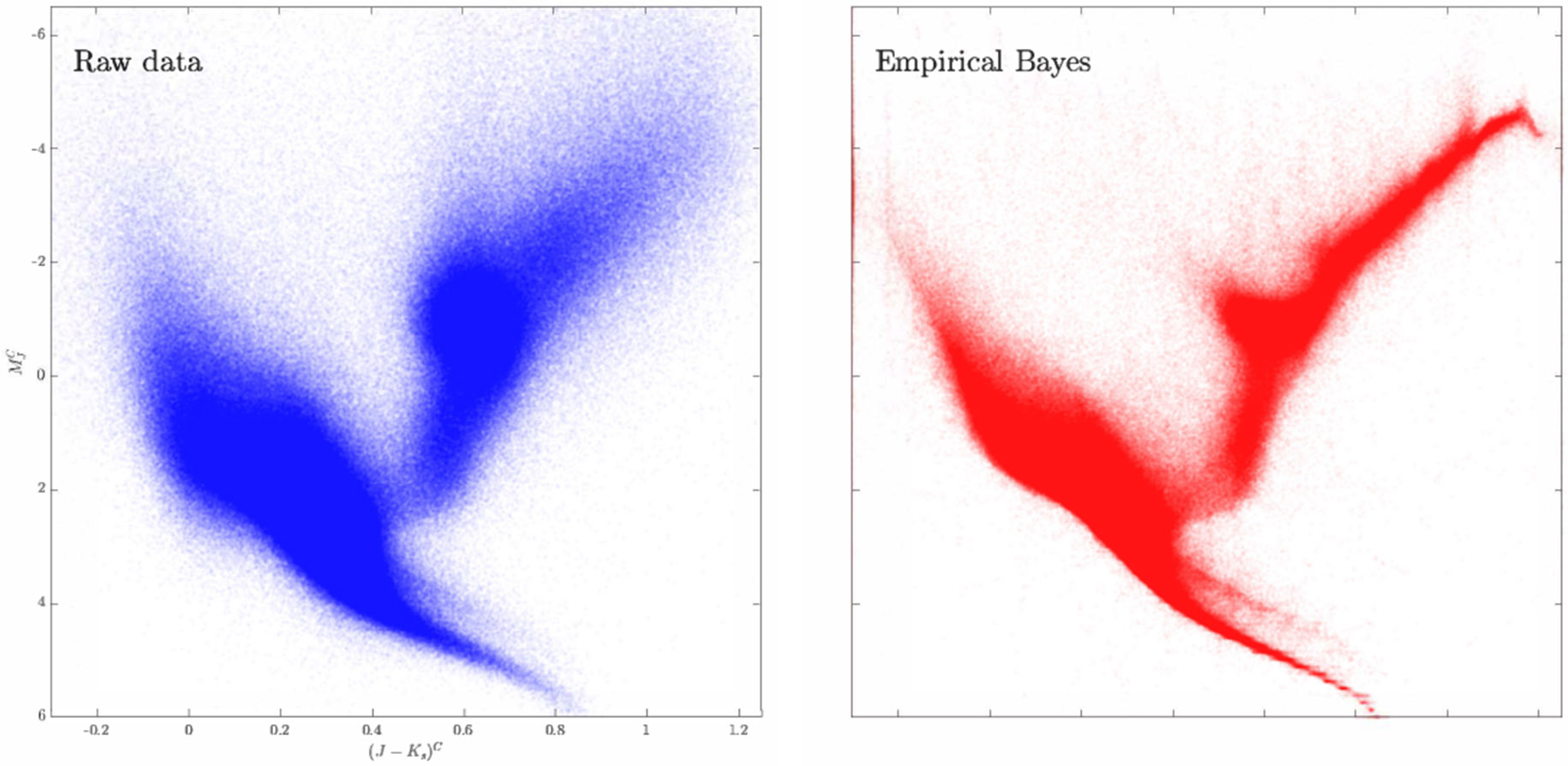
Left: The noisy CMD corresponding to data ${\left\{Y_{i}\right\}}_{i=1}^{n}\subset R^{2}$ for $n\approx 1.4\times 10^{6}$ stars obtained from the Gaia-TGAS Catalog. Right: The denoised CMD using empirical Bayes estimates ${\left\{{\hat{\theta }}_{i}\right\}}_{i=1}^{n}$ based on the NPMLE computed via the *augmented Lagrangian* method. The denoised CMD has rather sharp tails in the bottom of the plots (i.e., the main sequence) and the top right (i.e., the tip of the red-giant branch) as well as a definitive cluster in the center-right (i.e., the red clump).

We observe data $Y_{1},\ldots ,Y_{n}$ in $R^{d}$ (for d≥1) from the heteroscedastic Gaussian location mixture model

(1) $$Y_{i}=\theta_{i}+Z_{i},\;\text{with}\;\theta_{i}\overset{iid}{∼}G^{*}\;\text{and}\;Z_{i}\overset{ind}{∼}𝒩(0,\Sigma_{i})$$

where the underlying (unknown) latent parameters ${\left\{\theta_{i}\right\}}_{i=1}^{n}$ are assumed to be drawn i.i.d. from a common unknown distribution $G^{*}$ on $R^{d}$, and ${\left\{\Sigma_{i}\right\}}_{i=1}^{n}$ is a collection of known d×d positive definite heteroscedastic covariance matrices; assume further that $\theta_{i}$ and $Z_{i}$ are independent for each i=1,…,n. It is of importance to nonparametrically estimate $G^{*}$ and the latent variables ${\left\{\theta_{i}\right\}}_{i=1}^{n}$ that are observed with errors. Such mixture models arise naturally in various applications (Carlin and Louis, 1996; Efron, 2010; Efron and Hastie, 2021), including in the analysis of astronomy data (Akritas and Bershady, 1996; Hogg et al., 2010; Kelly, 2012); see the left panel of Figure 1 which shows the noisy color-magnitude diagram (CMD) corresponding to observations ${\left\{Y_{i}\right\}}_{i=1}^{n}$ for $n\approx 1.4\times 10^{6}$ stars from the Gaia-TGAS Catalog (Anderson et al., 2018).

Observe that the marginal density of $Y_{i}$ in (1) is given by

(2) $$f_{G^{*},\Sigma_{i}}(y)≔\int \phi_{\Sigma_{i}}(y-\theta )\;dG^{*}(\theta ),\;\;\text{for}\;y\in R^{d},$$

where $\phi_{\Sigma_{i}}(y)≔{\left[\text{det}(2\pi \Sigma_{i})\right]}^{-1∕2}\text{exp}(-y^{T}\Sigma_{i}^{-1}y∕2)$ is the density function of $𝒩(0,\Sigma_{i})$; further the observed $Y_{i}$’s are independent. A classical approach to estimating the unknown probability distribution $G^{*}$ in (1), which goes back to the works of Robbins (1950) and Kiefer and Wolfowitz (1956), is via the following *nonparametric maximum likelihood estimator* (NPMLE) which maximizes the marginal likelihood of the observations $Y_{i}$’s (Jiang and Zhang, 2009; Kiefer and Wolfowitz, 1956; Lindsay, 1983, 1995; Robbins, 1950):

(3) $${\hat{G}}_{n}\in \underset{G\in 𝒢}{\text{arg}\;\max}\frac{1}{n}\sum_{i=1}^{n}\log\;f_{G,\Sigma_{i}}(Y_{i}),$$

where the set 𝒢 consists of all probability distributions on $R^{d}$. Based on the solution ${\hat{G}}_{n}$ of (3), the marginal density $f_{G^{*},\Sigma_{i}}$ of $Y_{i}$ can be estimated by $f_{{\hat{G}}_{n},\Sigma_{i}}$, and each observation $Y_{i}$ can be *denoised* via the empirical Bayes estimator (see e.g., Soloff et al. (2021)):

(4) $${\hat{\theta }}_{i}≔E_{{\hat{G}}_{n}}[\theta_{i}\;|\;Y_{i}],\;\;\;\text{where}\;\theta_{i}∼{\hat{G}}_{n}\;\text{and}\;Y_{i}\;|\;\theta_{i}∼𝒩(\theta_{i},\Sigma_{i}),$$

to obtain an “estimate” of the underlying latent parameter $\theta_{i}$; see e.g., Efron (2019), Jiang and Zhang (2009), Soloff et al. (2021). For the noisy CMD from Anderson et al. (2018), the right panel of Figure 1 shows the denoised empirical Bayes estimate ${\left\{{\hat{\theta }}_{i}\right\}}_{i=1}^{n}$ based on the NPMLE solved via the *augmented Lagrangian* method proposed and studied in this paper.

We can see that the density function $f_{G,\Sigma_{i}}(\cdot )$ defined by (2) is linear in G and thus the objective function in (3) is concave in G (due to the concavity of the log function). Moreover, the domain of the variable G is the infinite dimensional space of all probability distributions 𝒢 which is a convex set. Thus, (3) is an *infinite dimensional convex optimization* problem that is challenging to solve computationally. Many numerical methods for approximately computing the NPMLE have been considered — including the expectation maximization (EM) algorithm (Laird, 1978), vertex direction and exchange methods (Böhning, 1985), semi-infinite methods (Lesperance and Kalbfleisch, 1992), constrained-Newton methods (Wang, 2007), and hybrid methods (Böhning, 2003; Liu and Zhu, 2007).

A natural way to alleviate the computational difficulty of (3) is to discretize (a compact region of) the whole space $R^{d}$ and restrict 𝒢 to the class of all distributions with a finite fixed support, say $\{\mu_{1},\ldots ,\mu_{m}\}\subseteq R^{d}$; see e.g., Koenker and Mizera (2014), Kim et al. (2020). Namely, we assume that every G∈𝒢 takes the form

(5) $$G=\sum_{j=1}^{m}x_{j}\delta_{\mu_{j}},\;\text{where}\;x_{j}\geq 0\;\forall j,\;\text{and}\;\sum_{j=1}^{m}x_{j}=1$$

for unknown mixture proportion $x={\left(x_{1},\ldots ,x_{m}\right)}^{T}$ and fixed $\left\{\mu_{1},\ldots ,\mu_{m}\right\}$ with m large; here by $\delta_{a}$ we mean the Dirac delta measure at a. Under the above reduction, (3) reduces to the following finite dimensional convex optimization problem:

(6) $$\underset{x={\left(x_{1},\ldots ,x_{m}\right)}^{T}\in R^{m}}{\mathrm{maximize}}\;\frac{1}{n}\sum_{i=1}^{n}\log\left(\sum_{j=1}^{m}L_{ij}x_{j}\right)\;\;\text{subject to}\;1_{m}^{T}x=1,\;x_{j}\geq 0\;\forall j,$$

where $L≔(L_{ij})\in R^{n\times m}$ is a fixed matrix with nonnegative entries such that $L_{ij}≔\phi_{\Sigma_{i}}(Y_{i}-\mu_{j})$ and $1_{m}$ denotes the vector of all ones in $R^{m}$. It can be shown that for every n, as m→∞, the optimal value of (6) converges to the optimal value of (3); see e.g., Royset and Wets (2022, Example 4.23 & Convergence 4.14). This justifies the discretization approach (5) to the infinite dimensional convex problem (3).

Observe that the optimization problem (6) can also arise in other contexts, e.g., it encompasses MLE for mixture proportions in a finite mixture model where the component densities are known. That is, suppose we observe $Y_{1},\ldots ,Y_{n}$ i.i.d. following the mixture density $\sum_{j=1}^{m}x_{j}f_{j}(\cdot )$ with unknown mixture proportion $x={\left(x_{1},\ldots ,x_{m}\right)}^{T}$ and known densities $f_{1},\ldots ,f_{m}$. Taking $L_{ij}=f_{j}(Y_{i})$, the MLE of x reduces to problem (6).

The most classical approach to NPMLE is the EM algorithm (Dempster et al., 1977). However, the EM may converge very slowly; see e.g., Redner and Walker (1984), Varadhan and Roland (2008), Koenker and Mizera (2014). Compared to the EM, modern convex optimization methods would be more efficient and stable. Among them, first-order methods, see e.g., Tran-Dinh et al. (2015), Dvurechensky et al. (2020), are natural choices for solving (6), although the convergence of first-order methods for solving this problem may slow down considerably as they approach the solution as shown by Kim et al. (2020, Section 4.3.5), especially when m and n are large. In principle, the convex problem (6) can also be solved by off-the-shelf interior point based solvers. In fact, the routine KWDual in the R package REBayes (Koenker and Gu, 2017) adopts the interior point method implemented by the commercial interior point solver MOSEK (Andersen and Andersen, 2000) to solve the Lagrangian dual formulation of (6). Although very stable and efficient for small to medium sized problems, the interior point method has inherently ill-conditioned normal equations that are extremely costly to solve by an iterative method when both m and n are large^1^. Recently, Wang et al. (2021) have proposed a cubic regularized Newton method to solve (6) for d=1 under additional shape constraints.

**Figure 2:**
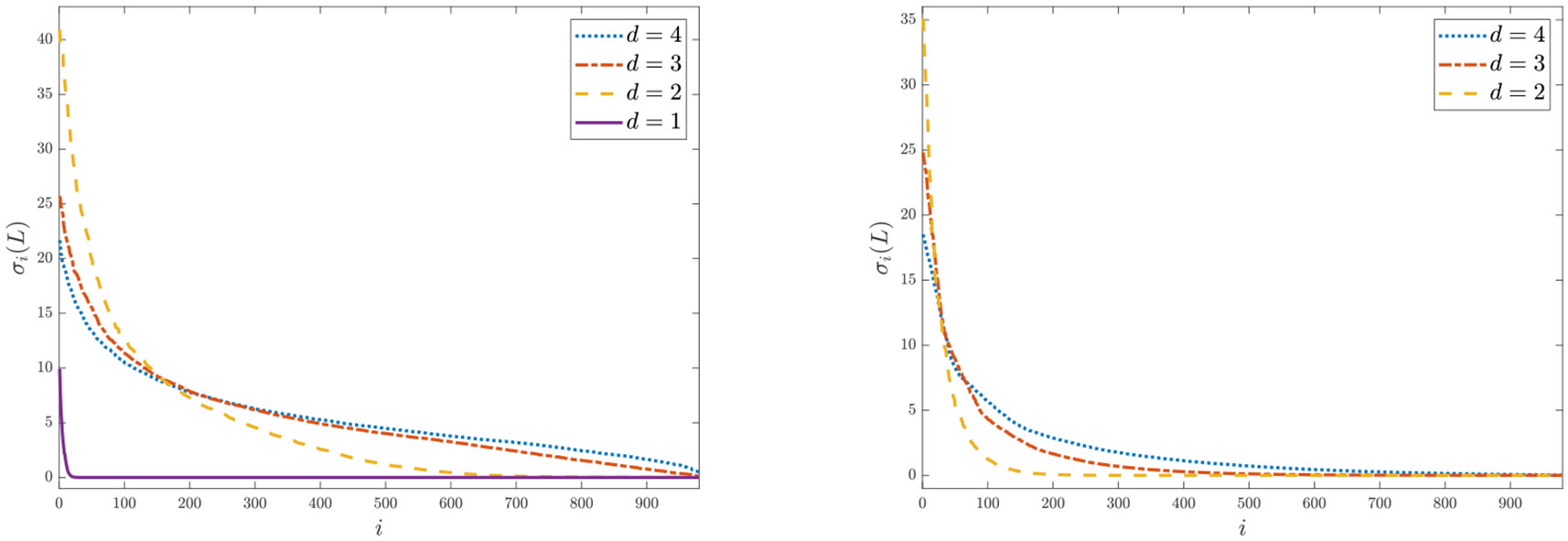
The distribution of the singular values $\sigma_{i}(L)$ of L computed from (left) the APOGEE data (here n=27, 135, and m=1,000) and (right) the synthetic Example 3(a) (here n=5,000, m=1,000) as d varies. The top 20 singular values are excluded so that the others are not overshadowed. Observe the slow decay of the singular values when d>1.

In order to solve (6) more efficiently an active set based sequential quadratic programming (SQP) method was recently proposed by Kim et al. (2020). The proposed algorithm mixsqp is able to solve (6) with large n (up to 10^6^) and small to medium m (up to several hundreds) very efficiently, by leveraging a low-rank approximation of the matrix L (in (6)) for univariate probability distributions (i.e., d=1). However, the low-rank approximation in the SQP method (Kim et al., 2020) may not work well for estimating multivariate (i.e., when d≥2) probability distributions $G^{*}$. Figure 2 shows the distributions of the singular values of the matrix L computed from the APOGEE survey (Majewski et al., 2017) (see Section 4.2 for details) and the synthetic Example 3(a) (see Appendix C.4 for details). We can see that most singular values of L are close to 0 when d=1, but L has a significant proportion of nonzero singular values when d≥2, and this phenomenon is more pronounced for larger d. This observation suggests that a low-rank approximation of L may lose crucial information in data fitting when d≥2. In addition, for problem (6), the number of grid points m needed to obtain a good approximation to the infinite dimensional problem (3) may be large for a large n, especially when d>1; see Figure 5 where we show plots for the noisy CMD data from Anderson et al. (2018) with different values of m.

The primary goal of the present paper is to provide a highly efficient, stable, and scalable numerical algorithm for solving problem (6) that can handle large n and m (e.g., $n\approx 10^{6}$ and $m\approx 10^{4}$). Our proposal is to apply the *augmented Lagrangian* method (ALM) for solving the dual problem of (6). Briefly, the ALM is an iterative method that solves a sequence of unconstrained subproblems to approximate the targeted constrained problem; see Hestenes (1969), Powell (1969), Rockafellar (1976). This method is expressed in terms of an augmented Lagrangian function. Let $L_{\sigma }(u,v;x,y)$ be the augmented Lagrangian function associated with the dual problem of (6), defined later in (9). Here (x, y) is the primal variable, (u, v) is the dual variable, and σ>0 is a positive parameter. For a nondecreasing sequence of parameters $\sigma_{k}>0$ and an initial primal variable ($x^{0}$, $y^{0}$), the ALM generates the primal iterative sequence {($x^{k}$, $y^{k}$)} and the dual iterative sequence {($u^{k}$, $v^{k}$)} as follows:

(7) $$(u^{k+1},v^{k+1})\approx \underset{u,v}{\text{argmin}}\;L_{\sigma_{k}}(u,v;x^{k},y^{k}),$$

(8) $$(x^{k+1},y^{k+1})=(x^{k},y^{k})+\sigma_{k}\nabla_{\left(x,y\right)}L_{\sigma_{k}}(u^{k+1},v^{k+1};x^{k},y^{k}),$$

where $\nabla_{\left(x,y\right)}L_{\sigma }$ denotes the gradient of $L_{\sigma }$ with respect to (x, y). Henceforth, problem (7) is referred to as the *ALM subproblem*. We call the ALM iterations as the outer loop, and the iterative method for solving the ALM subproblem as the inner loop. Details of the inner loop for solving the ALM subproblem are given in Section 2.1.

In Section 2.1, we show that the ALM subproblem (7) can be transformed into an unconstrained minimization problem of a continuously differentiable objective function, with the aid of the powerful tool of Moreau-Yosida regularization; see e.g., Rockafellar and Wets (2009, Chapter 1.G). This transformation allows us to work with a continuously differentiable objective function. Further, the gradient of this objective function is semismooth, although it is not smooth. Consequently, solving the ALM subproblem is equivalent to finding a solution to a semismooth equation. To tackle this, we can employ the *semismooth Newton* method; see e.g., Facchinei and Pang (2007, Chapters 7 and 8). To reduce the computational burden of the semismooth Newton method, our main technique is to exploit the sparsity in the corresponding generalized Hessian matrix (a nonsmooth counterpart of the Hessian matrix that arises in a second-order optimization problem; see (13) for details) leveraging the sparsity of the solution x (Koenker and Gu, 2017; Polyanskiy and Wu, 2020).

Although the sparsity of the solution x is also exploited by Kim et al. (2020) in computing the search direction for each SQP subproblem, their Hessian matrix itself is dense and the computational cost in evaluating each Hessian is $O((n+m)k^{2}+m^{2}k)$, with k being the (approximate) rank of the matrix L. In contrast, the generalized Hessian matrix arising from our semismooth Newton method is inherently sparse and the computational cost can be substantially reduced to O(nsmin(n,s)), where s is the number of nonzero elements in a certain vector in $R^{m}$ closely related to x; see (13) and the associated discussion. When s<n (in fact, s≪n in most situations; see Polyanskiy and Wu (2020)), the computational cost is $O(ns^{2})$; see Section 2.3 for details. The mixsqp method (Kim et al., 2020) demonstrates high efficiency when dealing with large n, moderate m, and numerically rank deficient L ($n\approx 10^{6}$, m ranging up to several hundreds, and k≪m). However, our algorithm can outperform mixsqp in two scenarios. Firstly, our algorithm exhibits better scalability in terms of m. Secondly, when L is no longer numerically rank deficient, our Hessian evaluation significantly reduces computational costs by fully leveraging sparsity.

Theoretically, we show that both the ALM for the outer loop (Algorithm 1) and the semismooth Newton method for the inner loop (Algorithm 2) have global convergence (see Proposition 1) and superlinear convergence rate (see Propositions 2 and 3). We illustrate the scalability and efficiency of our proposed method via extensive numerical experiments. In particular, as far as we are aware, for the 2-dimensional noisy CMD data set in the left panel of Figure 1, our ALM is the only known convex optimization method that can handle $m\approx 10^{4}$ grid points (with $n\approx 1.4\times 10^{6}$). For such a large m, even if we randomly subsample $n=10^{5}$ data points to make the package REBayes (Koenker and Gu, 2017) applicable (the mixsqp solver still does not work), the latter package is about 15 times slower than our ALM; see Section 4.2 for a comparison of these methods.

The second main contribution of this paper is related to the denoising of the observations ${\left\{Y_{i}\right\}}_{i=1}^{n}$. In (4), we have highlighted denoising the $Y_{i}$’s by the empirical Bayes estimator. In Section 3 we argue that these empirical Bayes estimates are not necessarily guaranteed to lie “close” to the support of $G^{*}$, which may be undesirable in certain applications. We propose new denoising estimands defined via the theory of optimal transport (Villani, 2003, 2009) that can mitigate this shortcoming of the empirical Bayes estimates. We also propose sample estimates of these new denoising estimands and prove, via a finite sample high probability bound (see Theorem 4), that the sample estimates are close to their population counterparts.

We conduct extensive numerical experiments on synthetic and real astronomy data sets in Section 4. We illustrate that, for problem (6), our ALM is much faster and scalable when compared to other existing solvers. Further, relevant codes for our methods, including simulation experiments, are available^2^. Some remarks, implementation details of the proposed methods, proofs of the main results, and extensive numerical experiments are relegated to the Appendix.

When d is large (e.g., d≥4), due to the curse of dimensionality, the choice of the support points $\mu_{j}$’s (see (5)) becomes important. A standard solution here is to let the support points also be variables, in which case the corresponding optimization problem (cf. (6)) becomes non-convex. Indeed this is what the famous EM algorithm (Dempster et al., 1977) solves; see Liu and Rubin (1994), Liu and Rubin (1998) for variants of this approach. In the online companion version of the current paper (Zhang et al. (2022)), we have also proposed a generalization of the EM approach — which we call as the partial EM algorithm — to solve the non-convex model that is provably convergent to its stationary point. Another line of approach for approximating (3) assumes G to belong to the class of finite Gaussian mixture models itself; see e.g., Bovy et al. (2011), Sarkar et al. (2018).

## 2. Augmented Lagrangian method for the dual of (6)

Since −log(⋅) is convex nonincreasing and scale invariant (i.e., for any t, α>0, −log(αt)=−logt−c for c≔logα), it has been observed that problem (6) is equivalent to the following convex problem with nonnegative constraints only (Kim et al., 2020, Proposition 3.2):

$$\underset{x={\left(x_{1},\ldots ,x_{m}\right)}^{T}\in R^{m}}{\mathrm{maximize}}\;\frac{1}{n}\sum_{i=1}^{n}\log\;\left(\sum_{j=1}^{m}L_{ij}x_{j}\right)-1_{m}^{T}x+1\;\text{subject to}\;x_{j}\geq 0,\;\forall j.$$

We introduce an auxiliary variable y to separate the components in the objective function and obtain the following primal problem:

(P) $$\underset{x\in R^{m},y\in R^{n}}{\mathrm{maximize}}\;\frac{1}{n}\sum_{i=1}^{n}\log\;y_{i}-1_{m}^{T}x+1\;\text{subject to}\;\frac{1}{n}(Lx-y)=0,\;x\geq 0.$$

Here by x≥0 we mean that every coordinate of x is nonnegative. To enhance the scaling of the problem and align with the factor $\frac{1}{n}$ in the objective function term $\sum_{i=1}^{n}\;\log\;y_{i}$, we introduce a scaling factor of $\frac{1}{n}$ for the equality constraint Lx−y=0. One can obtain the Lagrangian dual problem^3^ (Nocedal and Wright, 2006, Chapter 12.9) of (P) by maximizing the Lagrangian function associated with (P), i.e.,

$$\underset{x\geq 0\in R^{m},y\in R^{n}}{\mathrm{maximize}}\;\frac{1}{n}\sum_{i=1}^{n}\log\;y_{i}-1_{m}^{T}x+1+\frac{1}{n}u^{T}(Lx-y),$$

where $u\in R^{n}$ is the Lagrange multiplier. The dual problem admits the following formulation:

(D) $$\underset{u,v\in R^{n}}{\text{minimize}}\;h(u)-≔\frac{1}{n}\sum_{i=1}^{n}\log\;u_{i}\;\text{subject to}\;\frac{1}{n}L^{T}v\leq 1_{m},\;u-v=0.$$

Here the auxiliary variable $v\in R^{n}$ is introduced to separate the difficulties in dealing with the −log(⋅) objective function and the inequality constraint simultaneously. Strong duality clearly holds for (P) and (D) since the Slater condition trivially holds for problem (P) by taking $x_{j}=1∕m$ for all j=1,…,m; see Bertsekas (2016, Proposition 4.3.9).

We are now able to introduce the ALM applied to the dual problem (D). The ALM was first proposed by Hestenes (1969) and Powell (1969) for equality-constrained nonlinear programs. The augmented Lagrangian function involves quadratic penalties on the violation of equality constraints, and the ALM converts the minimization of an equality constrained problem into the minimization of a sequence of unconstrained problems. For a general convex nonlinear program (having both equality and inequality constraints), we can follow Rockafellar (1976) for the derivation of the ALM. For problem (D) having an inequality constraint $\frac{1}{n}L^{T}v\leq 1_{m}$, the augmented Lagrangian function (Rockafellar, 1976, (1.4)) is (here ${\left\|\cdot \right\|}_{2}$ denotes the usual Euclidean norm)

(9) Lσ(u,v;x,y)≔h(u)+yT(u−v)+σ2‖u−v‖22+{xT(1nLTv−1m)+σ2‖1nLTv−1m‖22if1nLTv−1m≥−xσ−12σ‖x‖22if1nLTv−1m≤−xσ}=h(u)+σ2‖max(1nLTv−1m+1σx,0)‖22−12σ(‖x‖22+‖y‖22)+σ2‖u−v+1σy‖22,

for σ>0 and a primal variable $(x,y)\in R^{m}\times R^{n}$; here max is a componentwise notation, and (9) is obtained by the completion of squares. The ALM for solving (D) contains two steps (7) and (8), and its algorithmic framework is given in Algorithm 1. The second step (8) is to update the Lagrange multipliers:

$$x^{k+1}=x^{k}+\sigma_{k}\nabla_{x}L_{\sigma_{k}}(u^{k+1},v^{k+1};x^{k},y^{k})=\max\left(\frac{\sigma_{k}}{n}L^{T}v^{k+1}-\sigma_{k}1_{m}+x^{k},\;0\right),\;y^{k+1}=y^{k}+\sigma_{k}\nabla_{y}L_{\sigma_{k}}(u^{k+1},v^{k+1};x^{k},y^{k})=y^{k}+\sigma_{k}(u^{k+1}-v^{k+1}).$$

The first expression for updating x follows from Rockafellar (1976, (1.8)), which can be obtained through the explicit computation of the gradient $\nabla_{x}L_{\sigma_{k}}$. The major computational cost in Algorithm 1 is to solve the ALM subproblem (7). Fortunately, the ALM subproblem can be transformed into an unconstrained minimization problem of a continuously differentiable objective function, as detailed in Section 2.1. It is worth noting that the gradient of this objective function is semismooth. This observation motivates us to employ a semismooth Newton method to solve the ALM subproblem (Li et al., 2018a,b; Zhang et al., 2020). It will exploit the special structure of the generalized Hessian of (9).

**Table:** 

Algorithm 1: An augmented Lagrangian method for solving (D)
Input:Data matrixL∈Rn×m;initial pointsx0∈Rm,y0∈Rn;penaltyparameterσ0>0;toleranceε≥0;ALM subproblem toleranceεk′≥0satisfying∑k≥0εk′<+∞;parameters for solving ALM subproblemη¯∈(0,1),τ∈(0,1],μ∈(0,1∕2),β∈(0.1).Output:xk,an approximate solution to(6).1fork=0,1,2,…do234567⌊(uk+1,vk+1)=SSN(L,xk,yk,σk,εk′22σk,η¯,τ,μ,β,0n);∕∕SolveALMsubproblem(7)viaAlgorithm2ifKKTresidual≤εthen⌊stop;∕∕StopiftheKKTresidul(42)isnomorethenεxk+1=max(σknLTvk+1−σk1m+xk,0);yk+1=yk+σk(uk+1−vk+1);Choose new penalty parmeterσk+1∈[σk,+∞);⌋

Finally, as suggested by one referee, there is an alternative approach to developing an ALM for solving (D). This approach introduces nonnegative slack variables to handle the inequality constraint and applies the “conventional” ALM used for equality-constrained problems; see e.g., Nocedal and Wright (2006, Chapter 17.3). We investigate this possibility in D and show that the alternative approach to developing ALM is equivalent to the ALM proposed in this section.

### 2.1 Semismooth Newton method for the ALM subproblem

To design the semismooth Newton method for solving the ALM subproblem, we first eliminate the variable u and transform the subproblem into a tractable continuously differentiable problem. To describe our approach we first review some basic concepts from convex analysis; see Appendix A.1.

Let $f\;:R^{n}\to (-\infty ,+\infty ]$ be a proper closed convex function. Parametrized by a scalar σ>0, the *Moreau-Yosida regularization* of f (also called *Moreau envelope* of f) and the (single-valued) proximal mapping of f at $x\in R^{n}$ are respectively defined as

$${ℳ}_{f}^{\sigma }(x)≔\underset{z\in R^{n}}{\text{min}}\left\{f(z)+\frac{\sigma }{2}{\left\|z-x\right\|}_{2}^{2}\right\}\;\text{and}\;{\text{Prox}}_{f}^{\sigma }\left(x\right)≔\underset{z\in R^{n}}{\text{argmin}}\left\{f(z)+\frac{\sigma }{2}{\left\|z-x\right\|}_{2}^{2}\right\}.$$

By dropping out the constant term $\frac{1}{2\sigma }({\left\|x\right\|}_{2}^{2}+{\left\|y\right\|}_{2}^{2})$ in (9), we can write the ALM subproblem ${\text{min}}_{u,v}L_{\sigma_{k}}(u,v;x^{k},y^{k})$ as

(10) $$\underset{u,v\in R^{n}}{\text{min}}\left\{h(u)+\frac{\sigma_{k}}{2}{\left\|\max\left(\frac{1}{n}L^{T}v-1_{m}+\frac{1}{\sigma_{k}}x^{k},0\right)\right\|}_{2}^{2}+\frac{\sigma_{k}}{2}{\left\|u-v+\frac{1}{\sigma_{k}}y^{k}\right\|}_{2}^{2}\right\}.$$

Note that the minimization in (10) with respect to u is achieved at $u^{*}≔{\text{Prox}}_{h}^{\sigma_{k}}(v-\sigma_{k}^{-1}y^{k})$ for any given v. Substituting $u^{*}$ back into (10) yields

$$\underset{v\in R^{n}}{\text{min}}\left\{\frac{\sigma_{k}}{2}{\left\|\max\left(\frac{1}{n}L^{T}v-1_{m}+\frac{1}{\sigma_{k}}x^{k},0\right)\right\|}_{2}^{2}+{ℳ}_{h}^{\sigma_{k}}(v-\sigma_{k}^{-1}y^{k})\right\}.$$

The above observation indicates that the ALM subproblem (7) can be achieved by a sequential update of v and u in the following way:

{vk+1≈argminv∈Rn{ϕk(v)≔σk2‖max(1nLTv−1m+1σkxk,0)‖22+ℳhσk(v−σk−1yk)},uk+1=Proxhσk(vk+1−σk−1yk).}

Therefore, the ALM subproblem is transformed into a tractable continuously differentiable problem ${\text{min}}_{v}\;\phi_{k}(v)$ since $\phi_{k}$ is convex and continuously differentiable, as both the squared max function ${\left\|\max(•,0)\right\|}^{2}$ and the Moreau envelope ${ℳ}_{h}^{\sigma_{k}}$ are continuously differentiable. As we know, to minimize a convex differentiable function, it suffices to set its gradient to zero. Therefore, we solve the problem ${\text{min}}_{v}\;\phi_{k}(v)$ via finding the solution of the following equation:

(11) $$\nabla \phi_{k}(v)=\frac{\sigma_{k}}{n}L\;\max\left(\frac{1}{n}L^{T}v-1_{m}+\frac{1}{\sigma_{k}}x^{k},0\right)+\sigma_{k}\left(v-\frac{1}{\sigma_{k}}y^{k}-{\text{Prox}}_{h}^{\sigma_{k}}\left(v-\frac{1}{\sigma_{k}}y^{k}\right)\right)=0,$$

where the gradient of the Moreau envelope ${ℳ}_{h}^{\sigma_{k}}$ is obtained via (33) in the Appendix.

Following the ALM discussed in the previous subsection, it is clear that the cornerstone of Algorithm 1 is the fast and scalable computation of (11). Due to the nonsmoothness of the componentwise max operation on the left side of (11), the classical Newton method for solving a smooth nonlinear equation may not be applicable here. Fortunately, the gradient $\nabla \phi_{k}$ is a so-called semismooth function (see Appendix A.1), in fact piecewise smooth, so that one may apply the *semismooth Newton* (see e.g., Facchinei and Pang (2007)) method to solve (11). It turns out that the nonsmoothness of this gradient equation is the key reason that our ALM is scalable.

The semismooth Newton method is a generalization of the classical Newton method for solving semismooth equations (Kojima and Shindo, 1986; Kummer, 1988; Qi and Sun, 1993). The basic idea of the former method is that for a semismooth function $F\;:R^{n}\to R^{m}$, one can still approximate F(x) locally at any given point $\bar{x}\in R^{n}$ by a linear mapping $F(\bar{x})+V(x-\bar{x})$ with residual $o(\|x-\bar{x}\|)$, where instead of taking $V=JF(\bar{x})$ as in the smooth case, we set V to be an arbitrary Clarke generalized Jacobian in the set ∂F(x); see Appendix A.1 for a review of these concepts.

Now, coming back to problem (11), the Clarke generalized Jacobian of the piecewise linear function $F_{\max}(x)=F_{\max}(x_{1},\ldots ,x_{m})≔(\max(x_{1},0),\ldots ,\max(x_{m},0))$ for $x\in R^{m}$ is given by

(12) $$\partial F_{\max}(x)=\{\text{Diag}(d)\;:d_{i}=1\;\text{if}\;x_{i}>0;\;d_{i}\in [0,1]\;\text{if}\;x_{i}=0,\;\text{and}\;d_{i}=0\;\text{if}\;x_{i}<0,\forall i\}\;.$$

Equipped with this Clarke generalized Jacobian, we can consider the following set-valued mapping as the collection of generalized Hessians of the function $\phi_{k}$:

(13) $$\partial^{2}\phi_{k}(v)=\left\{\sigma_{k}\left[\frac{1}{n^{2}}LSL^{T}+\underset{\text{denoted}\;D^{k}}{\underset{︸}{I_{n}-\nabla {\text{Prox}}_{h}^{\sigma_{k}}\left(v-\frac{y^{k}}{\sigma_{k}}\right)}}\right]:S\in \partial F_{\max}\left(\frac{1}{n}L^{T}v-1_{m}+\frac{x^{k}}{\sigma_{k}}\right)\right\}.$$

Two critical remarks on the set of generalized Hessians are in order. One, by noticing that

$$\;{\text{Prox}}_{h}^{\sigma }(y)=\frac{1}{2}\text{Diag}\left(y_{1}+\sqrt{y_{1}^{2}+4∕(\sigma n)},\ldots ,y_{n}+\sqrt{y_{n}^{2}+4∕(\sigma n)}\right),\;\nabla {\text{Prox}}_{h}^{\sigma }(y)=\frac{1}{2}I_{n}+\frac{1}{2}\text{Diag}\left(y_{1}∕\sqrt{y_{1}^{2}+4∕(\sigma n)},\ldots ,y_{n}∕\sqrt{y_{n}^{2}+4∕(\sigma n)}\right),$$

we get that $D^{k}$ in (13) is an n×n positive definite diagonal matrix. Two, one can derive from (12) that each $S\in \partial F_{\max}(L^{T}v∕n-1_{m}+x^{k}∕\sigma_{k})$ is an m×m diagonal matrix with either 0 or 1 in the diagonal entries. Let $s≔|\{i\;:S_{ii}=1\}|$, which represents the number of ones in S. Notably, while s depends on the current iterate $x^{k}$, v, and parameter $\sigma_{k}$, we have observed that it eventually aligns with the sparsity of the solution x. It is worth noting that if the dual variable v is feasible (which it generally is near the optimal solution), then $\frac{1}{n}L^{T}v\leq 1_{m}$. Consequently, the number of positive entries in $\max(\frac{1}{n}L^{T}v-1_{m}+{\sigma_{k}}^{-1}x^{k},0)$ cannot exceed the number of positive entries in $x^{k}$. As the primal solution x is usually sparse and the sequence $x^{k}$ gradually converges to x, one may expect that s will be relatively small during the semismooth Newton iterations, especially as we approach the optimal solution.

In fact, eventually s aligns with the sparsity of the solution x. The above two facts together indicate that the elements in $\partial^{2}\phi_{k}(v)$ are always positive definite and potentially have sparse structures — this is referred to as *second-order sparsity*. When $\partial^{2}\phi_{k}(v)$ consists of more than one matrix, we always take the sparsest one in our implementation.

The detailed steps of the semismooth Newton method for ${\text{min}}_{v}\;\phi_{k}(v)$ (i.e., solving $\nabla \phi_{k}(v)=0$ in (11)) is presented in Algorithm 2. Similar to the Newton method for solving smooth nonlinear equations, the semismooth Newton method with the unit step length only works locally near the solution. In order to make sure that the overall algorithm converges, we adopt the standard line search strategy in the second step of Algorithm 2 as the search direction computed from the first step of Algorithm 2 is always a descent direction of the objective function $\phi_{k}$; for details see Facchinei and Pang (2007, Section 8.3.3). We shall prove the convergence and the superlinear convergence rate of the generated sequence ${\left\{v^{t}\right\}}_{t\geq 1}$ in the next subsection.

**Table:** 

Algorithm 2: SSN(L, x^k^, y^k^, σk, ε, η¯, τ, μ, β, v^0^): A semismooth Newton method for ALM subproblem
Input:Data matrixL∈Rn×m;current iteratexk∈Rm,yk∈Rn;penaltyparameterσk>0;toleranceε≥0;η¯∈(0,1),τ∈(0,1](parameters forinexactness of the linear system);μ∈(0,1∕2),β∈(0,1)(parameters forbacktracking line search);initial pointv0∈Rn.Output:(ut,vt),an approximate solution to(7).1fort=0,1,2,…do23456789⌊if‖∇ϕk(vt)‖2>εthen⌊stop;ChooseHt∈∂2ϕk(vt)via(13);Compute a search directiondtby (approximately) solving the linear systemHtd=−∇ϕk(vt)(14)such that‖Htdt+∇ϕk(vt)‖2≤min(η¯,‖∇ϕk(vt)‖21+τ);αt=1;whileϕk(vt+αtdt)>ϕk(vt)+μαt〈∇ϕk(vt),dt〉do⌊αt=βαt;∕∕backtrackinglinesearchvt+1=vt+αtdt;⌋10ut≔Proxhσk(vt−σk−1yk);

### 2.2 Convergence results for the ALM and the semismooth Newton method

We now provide convergence guarantees and rates for both the ALM and the semismooth Newton method. Let ($\bar{u}$, $\bar{v}$) be an optimal solution of (D), i.e., there exists $(\bar{x},\bar{y})\in R^{n+m}$ such that the following Karush-Kuhn-Tucker (KKT) optimality conditions hold:

(15) {Lx¯=y¯,x¯≥0,(1nLTv¯−1m)Tx¯=0,1nLTv¯≤1m,u¯−v¯=0,andu¯i>0,u¯iy¯i=1,fori=1,…,n.}

In the seminal work of Rockafellar (1976), the global convergence and the asymptotically superlinear convergence rate of the ALM for solving convex problems were derived under the following two stopping criteria:

(S1) $$L_{\sigma_{k}}(u^{k+1},v^{k+1};x^{k},y^{k})-\underset{u,v\in R^{n}}{\text{inf}}L_{\sigma_{k}}(u,v;x^{k},y^{k})\leq \frac{\varepsilon_{k}^{2}}{2\sigma_{k}},$$

(S2) $$L_{\sigma_{k}}(u^{k+1},v^{k+1};x^{k},y^{k})-\underset{u,v\in R^{n}}{\text{inf}}L_{\sigma_{k}}(u,v;x^{k},y^{k})\leq \frac{\eta_{k}^{2}}{2\sigma_{k}}{\left\|(x^{k+1},y^{k+1})-(x^{k},y^{k})\right\|}_{2}^{2},$$

where ${\left\{\varepsilon_{k}\right\}}_{k\geq 0}$ and ${\left\{\eta_{k}\right\}}_{k\geq 0}$ are two prescribed positive summable sequences satisfying

(16) $$\max\left(\sum_{k=0}^{\infty }\varepsilon_{k},\sum_{k=0}^{\infty }\eta_{k}\right)<+\infty .$$

The positiveness of $\varepsilon_{k}$ and $\eta_{k}$ allows for inexact computation of the ALM subproblems. In practice, one may choose $\varepsilon_{k}=\eta_{k}=\beta^{-k}$ for some β>1. Under (S1) we will show (in Proposition 1 below) that the sequence ${\left\{(x^{k},y^{k})\right\}}_{k\geq 1}$ is convergent. This further implies that ${\text{lim}}_{k\to \infty }{\left\|(x^{k+1},y^{k+1})-(x^{k},y^{k})\right\|}_{2}=0$ so that the stopping criterion (S2) is in fact stronger than (S1). This stronger criterion yields a convergence rate for ${\left\{(x^{k},y^{k})\right\}}_{k\geq 1}$ (see Proposition 2).

Notice that the Slater condition trivially holds for problem (P) by taking $x_{j}=1∕m$ for all j=1,…,m so that a KKT solution ($\bar{u}$, $\bar{v}$, $\bar{x}$, $\bar{y}$) (satisfying the KKT conditions (15)) always exists; see Bertsekas (2016, Proposition 4.3.9). The following proposition regarding the global convergence of the sequence generated by the ALM is a consequence of Rockafellar (1976, Theorem 4).

**Proposition 1** Let ${\left\{\sigma_{k}\right\}}_{k\geq 0}$ be a nondecreasing positive sequence converging to $\sigma_{\infty }\leq \infty$. Let ${\left\{(u^{k},v^{k},x^{k},y^{k})\right\}}_{k\geq 1}$ be the sequence generated by Algorithm 1 with each subproblem satisfying the stopping criterion (S1). Then the primal sequence ${\left\{(x^{k},y^{k})\right\}}_{k\geq 1}$ converges to a solution ($\bar{x}$, $\bar{y}$) that solves problem (P).

Next we discuss the convergence rate of the ALM. Recall that a sequence ${\left\{w^{k}\right\}}_{k\geq 1}$ in $R^{n}$ is said to converge to $\bar{w}$ (with $w^{k}\neq \bar{w}$ for all k) *superlinearly* if ${\text{lim}}_{k\to \infty }{\left\|w^{k+1}-\bar{w}\right\|}_{2}∕{\left\|w^{k}-\bar{w}\right\|}_{2}=0$. The superlinear convergence rate of the ALM has been extensively studied in the existing literature since the pioneering work of Powell (1969). For convex nonlinear programming, the convergence rate of ${\left\{(x^{k},y^{k})\right\}}_{k\geq 1}$ can be derived under the so-called quadratic growth condition of problem (D) (Rockafellar, 1976; Cui et al., 2017). Recall that h(⋅) is the objective function of the dual problem defined in (D). Let ($\bar{u}$, $\bar{v}$) be the optimal solution of (D), which must be unique since h is strictly convex and $\bar{u}=\bar{v}$ due to the constraints. The quadratic growth condition of problem (D) pertains to the existence of a positive scalar κ and a neighborhood 𝒩 of ($\bar{u}$, $\bar{v}$) such that for any dual feasible solution (u,v)∈𝒩, satisfying $\frac{1}{n}L^{T}v\leq 1_{m}$ and u=v, the following inequality holds:

(17) $$h(u)\geq h(\bar{u})+\kappa \left({\left\|u-\bar{u}\right\|}_{2}^{2}+{\left\|v-\bar{v}\right\|}_{2}^{2}\right).$$

We show in the next result (see Appendix B.1 for a proof) that problem (D) satisfies this requirement, and thus the ALM for solving (D) has superlinear convergence rate.

**Proposition 2** Let ${\left\{\sigma_{k}\right\}}_{k\geq 0}$ be a nondecreasing positive sequence converging to $\sigma_{\infty }\leq \infty$. Let ${\left\{(u^{k},v^{k},x^{k},y^{k})\right\}}_{k\geq 1}$ be the sequence generated by Algorithm 1 with each subproblem satisfying the stopping criterion (S2), and ($\bar{x}$, $\bar{y}$) be the optimal solution of (P). Then either the algorithm converges in finite steps, or

$$\frac{{\left\|(x^{k+1},y^{k+1})-(\bar{x},\bar{y})\right\|}_{2}}{{\left\|(x^{k},y^{k})-(\bar{x},\bar{y})\right\|}_{2}}\leq \left(\frac{\kappa }{\sqrt{\kappa^{2}+\sigma_{k}^{2}}}+\eta_{k}\right){\left(1-\eta_{k}\right)}^{-1}.$$

The above proposition states that when the ALM subproblem is solved approximately under criterion (S2), the sequence ($x^{k}$, $y^{k}$) converges to an optimal pair ($\bar{x}$, $\bar{y}$) at a linear rate $(\kappa ∕\sqrt{\kappa^{2}+\sigma_{k}^{2}}+\eta_{k}){\left(1-\eta_{k}\right)}^{-1}$. Since $\eta_{k}\to 0$ due to (16) and $\sigma_{k}>0$, we know that the rate $(\kappa ∕\sqrt{\kappa^{2}+\sigma_{k}^{2}}+\eta_{k}){\left(1-\eta_{k}\right)}^{-1}$ is smaller than 1 when k is sufficiently large. In addition, as $\sigma_{k}\to \sigma_{\infty }$ as k→∞, the rate eventually converges to $\kappa ∕\sqrt{\kappa^{2}+\sigma_{\infty }^{2}}$. It is, roughly speaking, inversely proportional to $\sigma_{\infty }$ if $\sigma_{\infty }$ is large. If $\sigma_{\infty }=\infty$, the convergence is superlinear. This is the reason that we say the ALM has asymptotically superlinear convergence rate.

Notice that in Algorithm 2, the semismooth Newton method is terminated when the condition ${\left\|\nabla \phi_{k}(v^{t})\right\|}_{2}\leq \varepsilon$ is met at some point $v^{t}$, whereas in Propositions 1 and 2, the convergence and rate of convergence hold under the criteria (S1) and (S2). In fact, it has been established by Cui et al. (2019) that these latter two criteria, which are based on the function values, can be implied by the former condition based on the norm of the gradient.

Finally, we provide the global convergence and the local convergence rate of the semismooth Newton method (Algorithm 2) discussed in Section 2.1. These are standard results and one may consult the monograph Facchinei and Pang (2007, Chapters 7 and 8) for the detailed proofs.

**Proposition 3** Let ${\left\{v^{t}\right\}}_{t\geq 1}$ be the sequence generated by Algorithm 2. Then ${\left\{v^{t}\right\}}_{t\geq 1}$ converges globally to the unique solution $v^{*}$ of (11). Furthermore, the convergence rate is superlinear, i.e., ${\text{lim}}_{t\to \infty }{\left\|v^{t+1}-v^{*}\right\|}_{2}∕{\left\|v^{t}-v^{*}\right\|}_{2}=0$.

### 2.3 Comparison of the computational cost for second-order methods

In this subsection, we compare the computational cost per iteration for three second-order methods for solving (6): our semismooth Newton based ALM, the interior point method (implemented in the REBayes package (Koenker and Gu, 2017)) and the SQP method (implemented in mixsqp (Kim et al., 2020)).

#### Semismooth Newton based ALM.

The most expensive step in our ALM is to find the semismooth Newton direction from the linear system (14). It follows from the expression of $\partial^{2}\phi_{k}(v)$ in (13) that the linear equation (14) takes the following abstract form:

(18) $$\left(D+LSL^{T}\right)d=\text{rhs},$$

where D is an n×n positive definite diagonal matrix, S is an m×m diagonal matrix with diagonal entries being either 0 or 1, and rhs is a given vector in $R^{n}$. Denote $J≔\{i\;:S_{ii}=1\}$ and

(19) s≔∣J∣,the cardinality of the setJ,

and write $L_{J}\in R^{n\times s}$ as the sub-matrix of L with columns in J. Based on the special diagonal structure of S, one have that

$$D+LSL^{T}=D+L_{J}L_{J}^{T}.$$

Therefore, the cost of evaluating the generalized Hessian matrix once via $D+L_{J}L_{J}^{T}$ is $O(n^{2}s)$. When s<n, one can also solve (18) via the following Sherman-Morrison-Woodbury formula:

$${\left(D+L_{J}L_{J}^{T}\right)}^{-1}=D^{-1}-D^{-1}L_{J}{\left(I_{s}+L_{J}^{T}D^{-1}L_{J}\right)}^{-1}L_{J}^{T}D^{-1}.$$

Therefore, it suffices to solve a reduced linear system with the coefficient matrix being $I_{s}+L_{J}^{T}D^{-1}L_{J}\in R^{s\times s}$. The cost of computing $I_{s}+L_{J}^{T}D^{-1}L_{J}$ is $O(ns^{2})$, which is smaller than the direct evaluation of the Hessian matrix when s<n. Notice that for both cases, the computational cost for solving the linear equation (18) is independent of m.

Each gradient evaluation $\nabla \phi_{k}(\cdot )$ needs O(nm) operations due to the multiplications of L and $L^{T}$ with vectors; see (11). Since the number of gradient evaluations is the total number of semismooth Newton iterations for all ALM subproblems, one may expect that such evaluations do not need to be done many times.

In fact, we can also incorporate a low-rank approximation of L, as in the mixsqp solver (see (21) below), if the rank of L is indeed small to further reduce the computational cost of our gradient evaluations. With such techniques, the cost of each gradient evaluation is $O((n+m)k+\text{min}{\left(n,m\right)}^{2})$, where k is the numerical rank of the matrix L. In addition, the cost of each Hessian evaluation is reduced to $O(n\;\text{min}{\left(k,s\right)}^{2})$. In practice, we have noticed that our second-order sparsity s tends to be relatively small, and the reduction in cost from $O(ns^{2})$ (when s<n) to $O(n\;\text{min}{\left(k,s\right)}^{2})$, achieved by the low-rank approximation, is typically modest. Therefore, we have chosen not to implement the low-rank approximation of L when computing Hessian matrices in our ALM.

#### Interior point method.

We have found from the source code of REBayes (Koenker and Gu, 2017) that it calls the exponential cone optimization^4^ in MOSEK to solve (D). In fact, problem (D) can be formulated equivalently as the following exponential cone optimization problem:

(20) $$\underset{t,u\in R^{n}}{\text{minimize}}\;-\frac{1}{n}\sum_{i=1}^{n}t_{i}\;\text{subject to}\;\frac{1}{n}L^{T}u\leq 1_{m},\;\;(t_{i},u_{i},1)\in K_{\text{exp}},i=1,\ldots ,n,$$

where $K_{\text{exp}}≔\text{closure}\{(x,y,z)\in R^{3}\;|\;z>0,y\geq z\;\text{exp}(x∕z)\}$ is the nonsymmetric exponential cone studied by Chares (2009); here closure(⋅) denotes the closure of a convex set. It is well known that the interior point method for (20) generally relies on a logarithmically homogeneous self-concordant barrier (LHSCB) (and its conjugate barrier) of the exponential cone (and its dual cone). Notice that the constraints of problem (20) involves n numbers of exponential cones. From Yuan (2017, Proposition 1.2.4), we can see that the cost of computing the gradient and Hessian of LHSCBs for all these exponential cones is O(n). The most expensive step in the interior point method is to find a search direction of a linear system (e.g., Yuan (2017, (2.2)), Dahl and Andersen (2022, (4))) for the central path. In particular, the Schur complement equation (e.g., Yuan (2017, (2.13))) of the linear system involves computing $L^{T}g$ and $L^{T}HL$, where $g\in R^{n}$ and $H\in R^{n\times n}$ are associated with the gradient and Hessian of the LHSCB. The cost of computing $L^{T}g$ and $L^{T}HL$ is O(nm) and $O(n^{2}m)$ respectively.

#### SQP.

For the SQP method implemented in the mixsqp solver (Kim et al., 2020), the gradient g and Hessian H for each SQP subproblem are given by

$$g=-\frac{1}{n}L^{T}d+1_{m}\;\text{and}\;H=\frac{1}{n}L^{T}\text{diag}{\left(d\right)}^{2}L,$$

where $d={\left(1∕{\left(Lx\right)}_{1},\cdots ,1∕{\left(Lx\right)}_{n}\right)}^{T}\in R^{n}$ for some given $x\in R^{m}$. Recall that L is an n×m matrix. The cost of naively computing the gradient and Hessian is O(nm) and $O(nm^{2})$ respectively. In the solver mixsqp, when the matrix L is numerically rank deficient, say rank ≈k, then the matrix L can be approximated by the following truncated QR decomposition (if m≤n):

(21) $$L\approx QRP^{T},\;\text{with}\;Q\in R^{n\times k},\;R\in R^{k\times m},\;P\in R^{m\times m}.$$

The cost of computing the gradient and Hessian in mixsqp then reduces to $O((n+m)k+\text{min}{\left(n,m\right)}^{2})$ and $O((n+m)k^{2}+m^{2}k)$ respectively.

The main comparison results are given in Table 1. The cost of each Hessian evaluation in the ALM is O(nsmin(n,s)). As elaborated in Section 2.1, eventually the second-order sparsity s is closely tied to the sparsity of the solution x. As we know, the solution x is usually very sparse (Koenker and Gu, 2017; Polyanskiy and Wu, 2020). In most situations, we have s≪n and the Hessian evaluation of the ALM requires $O(ns^{2})$ operations. This is a substantial reduction compared to the $O(nm^{2})$ cost of each Hessian evaluation in mixsqp when the matrix L is no longer numerically low rank. The table shows how the second-order sparsity helps to reduce the computational burden in our approach.

## 3. Denoising via optimal transport

In this section we consider the Gaussian location mixture model (1) and present new denoising *estimands* defined through a *matching* idea (via the theory of optimal transport (Villani, 2003, 2009)). To motivate our proposal, let us first describe the rationale behind (4). The problem of denoising the observed $Y_{i}$’s can be formally described using the following Bayesian framework. It is known that if the goal is to minimize the expected squared error *Bayes risk*

(22) $$E\;[{\left\|\vartheta (Y_{i})-\theta_{i}\right\|}_{2}^{2}\equiv \int \int {\left\|\vartheta (y)-\theta \right\|}_{2}^{2}\;\phi_{\Sigma_{i}}(y-\theta )\;dG^{*}(\theta )\;dy$$

over all measurable functions $\vartheta \;:R^{d}\to R^{d}$, where $\theta_{i}∼G^{*}$ and $Y_{i}\;|\;\theta_{i}∼𝒩(\theta_{i},\Sigma_{i})$, then the best estimator for $\theta_{i}$ is the *oracle posterior mean*

(23) $$\vartheta^{*}(Y_{i})≔E[\theta_{i}\;|\;Y_{i}].$$

**Figure 3:**
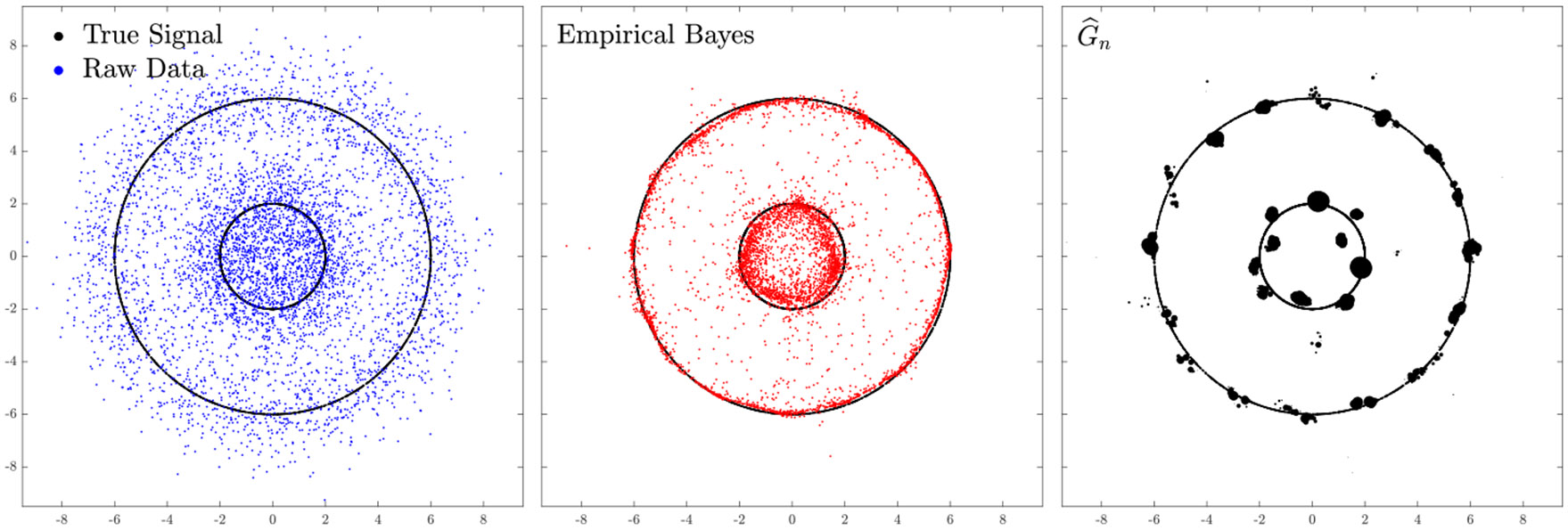
Plots of the raw data (in blue) with n=5,000 in d=2, the corresponding empirical Bayes estimates (in red), the true $G^{*}$ (in black), and ${\hat{G}}_{n}$ (in black dots) obtained from our ALM. Here half of the true signals $\theta_{i}\in R^{d}$ are drawn uniformly at random from each of the two concentric circles of radii 2 and 6 respectively (centered at $(0,0)\in R^{2}$), and $Y_{i}\;|\;\theta_{i}∼𝒩(\theta_{i},I_{2})$ for i=1,…,n. Observe that some of the empirical Bayes estimates ${\hat{\theta }}_{i}$’s are far from the support of $G^{*}$ (and ${\hat{G}}_{n}$)

In empirical Bayes, given an estimate ${\hat{G}}_{n}$ of the unknown prior $G^{*}$, one imitates the optimal Bayesian analysis and estimates the oracle posterior means by the empirical Bayes estimates (4); see e.g., Jiang and Zhang (2009), Efron (2019). Although this is a natural strategy which has been studied extensively in the literature, there are a few drawbacks of this plug-in approach:

(1) The *oracle posterior mean* $\vartheta^{*}(Y_{i})$ in (23) and the empirical Bayes estimates in (4) are not necessarily lying “close” to the support of $G^{*}$ (say $𝒮\subset R^{d}$). In fact, if the goal is to estimate $\theta_{i}∼G^{*}$, it is reasonable to restrict ϑ(⋅) in (22) to all estimators such that $\vartheta (Y_{i})$ is distributed (approximately) as $G^{*}$. To illustrate this phenomenon suppose that $G^{*}$ has *structure* (e.g., the $\theta_{i}$’s are supported on a lower dimensional manifold 𝒮, or $G^{*}$ is discrete with few atoms which corresponds to the clustering problem); see e.g., Figure 3. The empirical Bayes estimator ${\hat{\theta }}_{i}$ in (4), may *not* necessarily, in general, lie “close” to the set 𝒮 (see the red points in the middle panel of Figure 3). Thus, if the emphasis is on estimating $\theta_{i}$’s focussing on recovering the support 𝒮, the estimates ${\hat{\theta }}_{i}$’s are not necessarily ideal.

(2) It is worth noting that although we call ${\hat{\theta }}_{i}$’s as natural estimates of $\theta_{i}$, they are not *consistent* estimates, in the sense that generally, ${\hat{\theta }}_{i}$ does not converge (e.g., in probability) to $\theta_{i}$.

To motivate our alternative approach, first suppose that $\Sigma_{i}\equiv \Sigma$ for all i=1,…,n and that the $\theta_{i}$’s are known up to a permutation, i.e., the empirical distribution

(24) $$G_{n}≔\frac{1}{n}\sum_{i=1}^{n}\delta_{\theta_{i}}$$

of the $\theta_{i}$’s is known. Then, it seems natural to associate $Y_{i}$ to a $\theta_{j}$ by solving the *matching* (optimization) problem: ${\text{min}}_{\sigma :[n]\to [n]}\frac{1}{n}\sum_{i=1}^{n}{\left\|Y_{i}-\theta_{\sigma (i)}\right\|}^{2}$, where σ=(σ(1),…,σ(n)) is a permutation of [n]≔{1,…,n}. In other words, we match the data points $Y_{i}$’s to the $\theta_{j}$’s such that the average cost of the matching is smallest. Letting $\nu_{n}≔\frac{1}{n}\sum_{i=1}^{n}\delta_{Y_{i}}$ denote the empirical distribution of the observed $Y_{i}$’s, this matching problem can be formulated as an *optimal transport* (OT) problem (see Appendix A.2 for a brief introduction):

(25) $$\underset{T:T\#\nu_{n}=G_{n}}{\text{min}}\frac{1}{n}\sum_{i=1}^{n}{\left\|Y_{i}-T(Y_{i})\right\|}_{2}^{2}$$

where the above minimization is over all maps T such that $T\#\nu_{n}=G_{n}$ which means that T transports the distribution of $\nu_{n}$ to $G_{n}$, i.e., $T\;:\{Y_{1},\ldots ,Y_{n}\}\to \{\theta_{1},\ldots ,\theta_{n}\}$ is a bijection. Note that (25) can be viewed as an *assignment problem* for which algorithms with worst case complexity $O(n^{3})$ are available in the literature (see e.g., Munkres (1957), Bertsekas (1988)). It is known that the minimum value of the above objective matches the Wasserstein (squared) distance between $\nu_{n}$ and $G_{n}$.

Problem (25) can be cast in the population setting by considering (cf. (22))

(26) $$\underset{𝒯\#\nu =G^{*}}{\text{min}}E\;[{\left\|Y-𝒯(Y)\right\|}_{2}^{2}]\equiv \underset{\pi \in \Pi (\nu ,G^{*})}{\text{min}}\int {\left\|y-\theta \right\|}^{2}\;d\pi (y,\theta )≕W_{2}^{2}(\nu ,G^{*})$$

over all functions $𝒯\;:R^{d}\to R^{d}$ such that $𝒯\#\nu =G^{*}$, which means that 𝒯 transports ν — the marginal distribution of Y — to $G^{*}$, i.e., $𝒯(Y)∼G^{*}$ where Y∼ν. The right side of (26) involves minimization over $\Pi (\nu ,G^{*})$ — the class of all joint distributions π with marginals ν and $G^{*}$, and gives the equivalence^5^ of Monge’s problem and Kantorovich’s relaxation (Villani, 2003, 2009).

Suppose that ${𝒯}^{*}$ is the optimal solution to (26); i.e., ${𝒯}^{*}$ is the OT map such that ${𝒯}^{*}\#\nu =G^{*}$. It is known from the theory of OT that such a ${𝒯}^{*}$ exists, is unique a.e., and can be expressed as the gradient of a convex function; see e.g., Villani (2003), Villani (2009). Then,

(27) $${\tilde{\theta }}_{i}≔{𝒯}^{*}(Y_{i}),\;\;\text{for}\;i=1,\ldots ,n,$$

could be considered as a natural *denoising* target for $Y_{i}$. Observe that, by (26), we have ${\tilde{\theta }}_{1},\ldots ,{\tilde{\theta }}_{n}$ are i.i.d. $G^{*}$ (compare this with the fact that $\theta_{1},\ldots ,\theta_{n}$ are also i.i.d. $G^{*}$). Further, the new estimand ${\tilde{\theta }}_{i}$ is related to $Y_{i}$ directly through the map ${𝒯}^{*}$ via (27). One can think of ${\tilde{\theta }}_{i}∼G^{*}$ as the “closest” (in the sense of distributions) to $Y_{i}∼\nu$. In the following we will consider estimation of ${\tilde{\theta }}_{1},\ldots ,{\tilde{\theta }}_{n}$ as defined in (27).

In order to estimate our new denoising targets ${\tilde{\theta }}_{1},\ldots ,{\tilde{\theta }}_{n}$ we first need to estimate ${𝒯}^{*}$, defined via (26). A natural plug-in approach here would be to replace ν and $G^{*}$ in (26) with $\nu_{n}$ (the empirical distribution of $Y_{1},\ldots ,Y_{n}$) and ${\hat{G}}_{n}$ (see (3)). Thus, we solve the linear program

(28) $$\underset{\pi \in \Pi (\nu_{n},{\hat{G}}_{n})}{\text{min}}\int {\left\|y-\theta \right\|}^{2}\;d\pi (y,\theta )\equiv W_{2}^{2}(\nu_{n},{\hat{G}}_{n}).$$

As ${\hat{G}}_{n}$ has finite support (see Soloff et al. (2021)), and $\nu_{n}$ is a discrete distribution, the optimal coupling in (28) can be represented by a matrix $\hat{\pi }={\left(({\hat{\pi }}_{ij})\right)}_{n\times \hat{k}}$ which has marginal $\nu_{n}$ and ${\hat{G}}_{n}$; here we suppose that ${\hat{G}}_{n}=\sum_{j=1}^{\hat{k}}{\hat{\alpha }}_{j}\delta_{{\hat{a}}_{j}}$, where ${\hat{a}}_{1},\ldots ,{\hat{a}}_{\hat{k}}\in R^{d}$ and ${\hat{\alpha }}_{j}$’s are positive weights summing up to 1. To obtain a transport map from this joint coupling $\hat{\pi }$ we can use the idea of *barycentric projection* (see Deb et al. (2021)) and define

(29) $${\hat{T}}_{n}(Y_{i})≔E_{\hat{\pi }}[\theta \;|\;Y_{i}]=n\sum_{j=1}^{\hat{k}}{\hat{\pi }}_{ij}{\hat{a}}_{j},$$

as an estimator of ${\tilde{\theta }}_{i}\equiv {𝒯}^{*}(Y_{i})$. As ${\hat{G}}_{n}$ is a discrete distribution with much fewer atoms than n, most of the $Y_{i}$’s will be essentially transported to one element in ${\hat{G}}_{n}$; see the right panel of Figure 3. Thus the estimates ${\hat{T}}_{n}(Y_{i})$ will essentially lie in the support of ${\hat{G}}_{n}$; this rectifies the drawbacks of the empirical Bayes approach outlined at the beginning of this section.

In the following result (proved in Appendix B.2) we show that our proposed estimand ${\tilde{\theta }}_{i}\equiv {𝒯}^{*}(Y_{i})$, in (27), can be consistently estimated by the estimator ${\hat{T}}_{n}(Y_{i})$ defined in (29). In fact, the above result provides a finite sample bound on the rate of convergence of ${\hat{T}}_{n}$ in average (squared) Euclidean norm.

**Theorem 4** Suppose that we have data from (1) where $\Sigma_{i}\equiv \Sigma$ for all i=1,…,n, and Σ is a d×d positive definite matrix with minimum eigenvalue σ>0. Suppose that the denoising estimands ${\tilde{\theta }}_{i}$’s are defined via (27) where ${𝒯}^{*}=\nabla \psi$ with $\psi \;:R^{d}\to R$ being a convex function. We assume that ψ is λ-strongly convex^6^ and L-smooth^7^, for λ, L>0, and that $G^{*}$ is compactly supported, i.e., $G^{*}({\left[-M,M\right]}^{d})=1$, for some M>0. Then there is a function n(d,σ,M) and a constant $C_{d,\sigma }>0$ such that, for all sample sizes n with n≥n(d,σ,M), with probability at least $1-\frac{4d}{n^{8}}$,

(30) $$\frac{1}{n}\sum_{i=1}^{n}{\left\|{\hat{T}}_{n}(Y_{i})-{\tilde{\theta }}_{i}\right\|}_{2}^{2}\leq C_{d,\sigma }\frac{L}{\lambda }\frac{1}{\log\;n}.$$

Our proof technique for Theorem 4 first relates the left-hand side of (30) to $W_{2}^{2}(G_{n},{\hat{G}}_{n})$ — the deconvolution error in the Wasserstein metric (see Deb et al. (2021), Manole et al. (2021)). It is well known that the smoothness of the Gaussian errors makes the deconvolution problem difficult; in fact, the logarithmic rate is minimax optimal for deconvolution with Gaussian errors (see e.g., Dedecker and Michel (2013), Soloff et al. (2021)).

## 4. Numerical experiments

In this section we illustrate the efficiency and scalability of our semismooth Newton based ALM via numerical experiments on both simulated and real data. We compare the ALM with the state-of-the-art R package mixsqp (Kim et al., 2020)^8^, and the R package REBayes (Koenker and Gu, 2017). The KWDual function in the latter package solves the dual formulation (D) by an interior point method using the commercial solver MOSEK (Andersen and Andersen, 2000).

All numerical experiments of our algorithm were performed in Matlab (version 9.11) on a Windows workstation (32-core, Intel Xeon Gold 6226R @ 2.90GHz, 128 GB of RAM), except explicitly mentioned otherwise. The R package mixsqp and the KWDual function in the R package REBayes were called in R 4.1.2. The KWDual function used version 9.3 of the MOSEK optimization library. Our methods implemented in Matlab and the relevant codes, including simulation experiments, are available at https://github.com/YangjingZhang/Dual-ALM-for-NPMLE.

Due to space constraints, in Appendix C.1 we provide a detailed discussion on the stopping criteria and implementation details of the methods used in our numerical experiments. In Appendix C.2, we conduct preliminary experiments comparing our method to first-order methods (e.g., a projected gradient method and a limited-memory projected quasi-Newton method). It provides insights into why we have excluded first-order methods from our main comparison.

### 4.1 One-dimensional synthetic data

We first present the numerical results on several one-dimensional synthetic data sets. The main purpose of these experiments is to show the efficiency and scalability of our ALM in terms of n (the number of observations) and m (the number of support points).

#### Example 1.

We replicate the simulation experiment conducted in Brown and Greenshtein (2009), Jiang and Zhang (2009), Johnstone and Silverman (2004). Consider n independent observations where $Y_{i}∼𝒩(\theta_{i},1)$, with each $\theta_{i}$ taking the value 0 or ν with the proportion of ν being τ=0.5%n, 5%n, or 50%n. We use equally spaced support points on the interval [${\text{min}}_{i}\;Y_{i}$, $\max_{i}\;Y_{i}$] for a given number of grid points m.

In Table 2, we report the numerical performance of our ALM, mixsqp and REBayes for relatively small-size instances with n=1,000 and m=500 (averaged over 10 replications). It can be seen from Table 2 that our ALM outperforms the other two methods for all instances — the ALM is the fastest algorithm which also produces the smallest KKT residual (given in (42)). From the last column “relative objective value” of Table 2, we can see that the three methods yield satisfactory solutions of comparable quality.

In order to illustrate the scalability of our ALM, we further repeat the experiment on large instances with n=10,000 and m=5,000. The corresponding results are recorded in Table 3. We do not include the results for mixsqp here since it takes an excessively long time for problems of this scale (where m>1,000). Here for one particular instance with n=10,000 and m=5,000, mixsqp took approximately 7 hours to complete 10 SQP iterations. However, it still failed to converge (with a residual exceeding 10^−2^). As shown in Table 3, the computational time of our ALM for each instance is less than 5 seconds, which is about 20 times faster than REBayes. In fact, about 3 seconds of our ALM are spent for the one-time computation of a low-rank approximation of the matrix L; the rest of the computation (including the computation of the gradients and the generalized Hessians as well as solving the semismooth Newton equations) is completed in 1 second.

#### Example 2.

We replicate the experiment conducted in Kim et al. (2020), where 50%, 20%, and 30% of the observations ${\left\{Y_{i}\right\}}_{i=1}^{n}$ are draw independently from 𝒩(0,1), $t_{4}$, and $t_{6}$ distributions respectively. Here $t_{\nu }$ denotes Student’s t-distribution with ν degrees of freedom. As the observed data can be modeled as a Gaussian *scale* mixture, we find ${\hat{G}}_{n}$ by solving a mixture problem of the form $\sum_{j=1}^{m}x_{j}g_{j}$, $x_{j}\geq 0$, $\sum_{j=1}^{m}x_{j}=1$, where $g_{j}$ is the density of $𝒩(0,\sigma_{j}^{2})$ for some given $\sigma_{j}$, j=1,…,m. Following Kim et al. (2020), we select the grid values $\left\{\sigma_{1}^{2},\ldots ,\sigma_{m}^{2}\right\}$ by the method in Stephens (2017).

**Figure 4:**
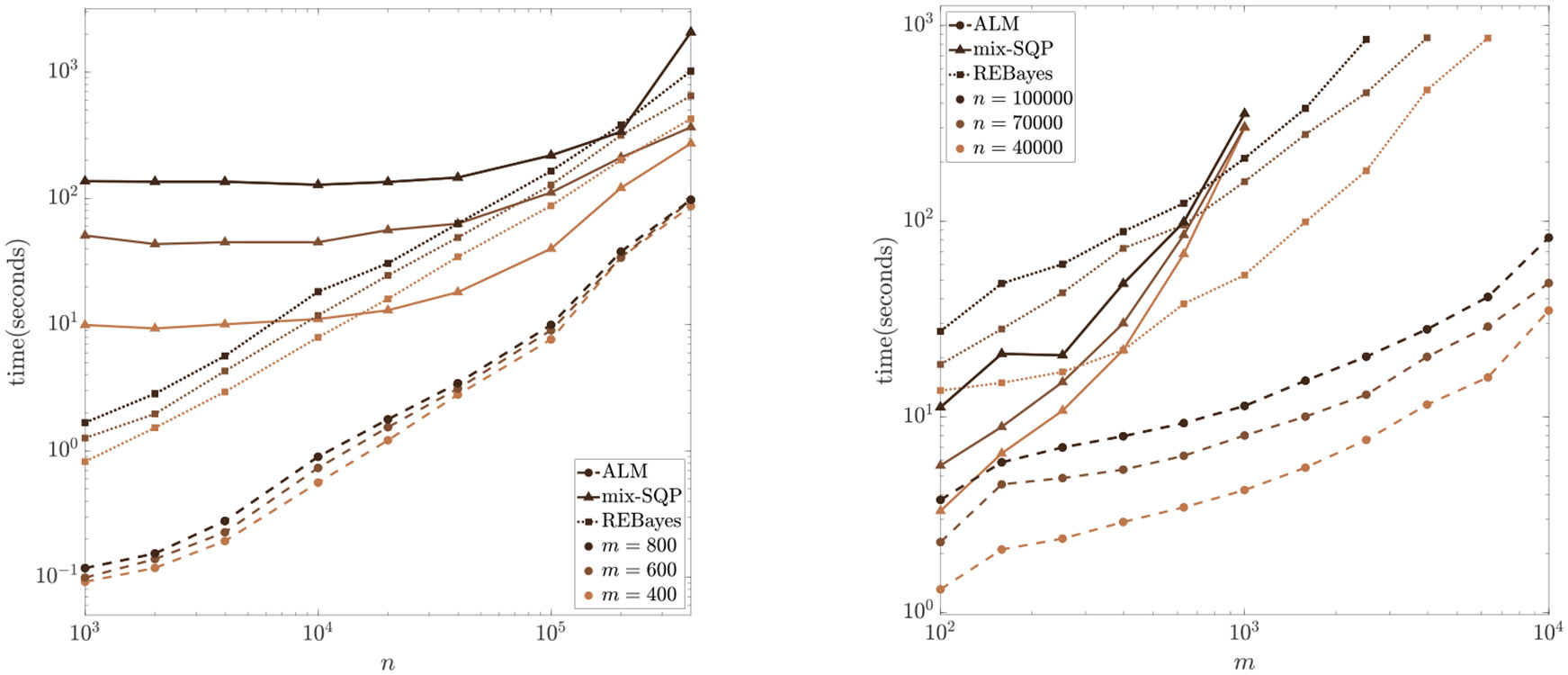
Computational time (in seconds) of the ALM, mixsqp, and REBayes with changing n and m for Example 2 (averaged over 10 replications).

We test the scalability of the ALM, mixsqp and REBayes for different values of n and m, and the results are shown in Figure 4. On the left panel, we consider m∈{400,600,800} and $n\in \text{ceil}\{10^{3},10^{3.3},10^{3.6},10^{4},10^{4.3},10^{4.6},10^{5},10^{5.3},10^{5.6}\}$. We consider even larger instances with $n\;\in \;\{4\;\times \;10^{4},7\;\times \;10^{4},10^{5}\}$ and $m\;\in \;\text{ceil}\{10^{2},10^{2.2},10^{2.4},10^{2.6},10^{2.8},10^{3},10^{3.2},10^{3.4},10^{3.6},10^{3.8},10^{4}\}$ on the right panel of Figure 4. We found that when m>1,000, mixsqp usually fails to solve the instances within 100 iterations under the stopping criterion $\varepsilon =10^{-6}$; as a result, we have not included the results for mixsqp when m>1,000 in the plot. Although the REBayes solver is able to solve most instances, it takes about 100 times more computational time compared to our ALM. In particular, for the largest test instance with $n=10^{5}$ and $m=10^{4}$ it only takes the ALM about 80 seconds to get a highly accurate solution. However, REBayes failed to solve this instance. In addition, to assess the quality of the solutions produced by the compared methods, we present the KKT residual (given in (42)) plotted against n and m in Figure 8 in the Appendix. Figures 4 and 8 reveal that our algorithm consistently achieves more accurate solutions, characterized by smaller KKT residuals, compared to the other two methods. Furthermore, our algorithm achieves this while requiring less computational time. Furthermore, the performance of all the methods remains consistently stable across various replications. Refer to Figure 9 in Appendix C.3 for a plot depicting the average computational times, along with error bars indicating the standard deviation across 10 replications.

### 4.2 Two-dimensional astronomy data

We analyze two astronomy data sets obtained from Gaia-TGAS (Brown et al., 2016) and APOGEE (Majewski et al., 2017).

#### Data set 1 (Gaia-TGAS).

**Figure 5:**
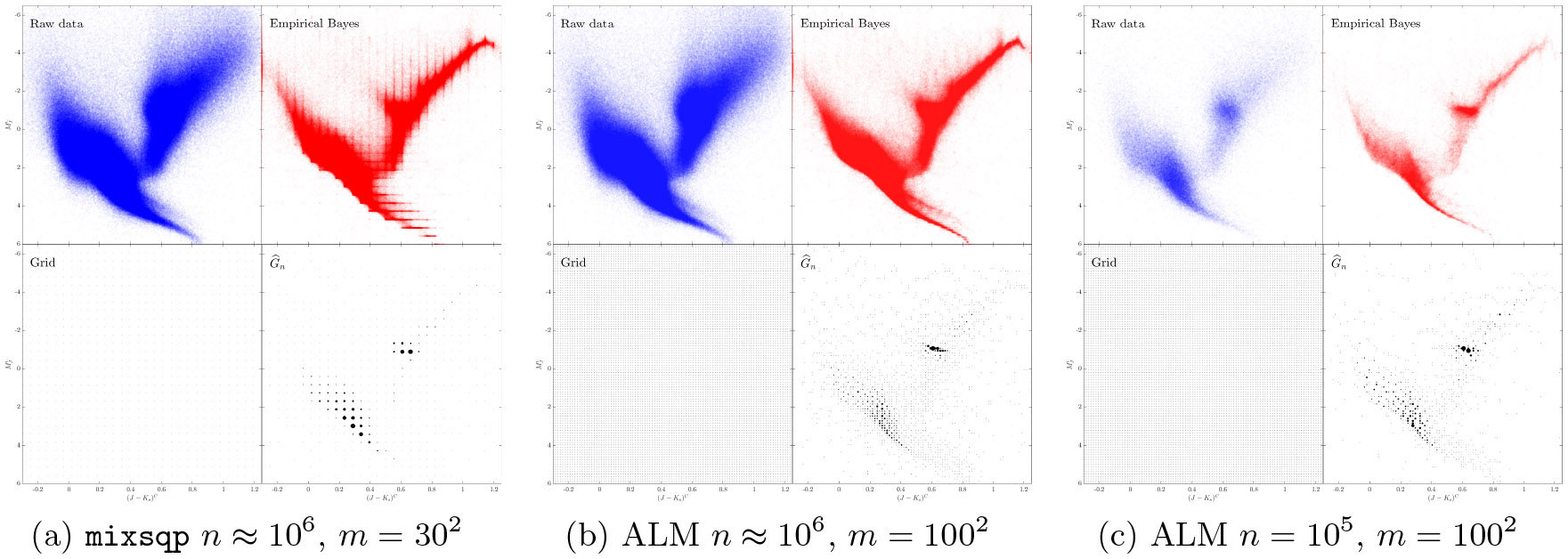
Results for the d=2 dimensional Gaia-TGAS data obtained from: (a) mixsqp (with $n\approx 10^{6}$, $m=30^{2}$), (b) ALM (with $n\approx 10^{6}$, $m=100^{2}$), (c) ALM (with $n=10^{5}$, $m=100^{2}$). The number of support points of ${\hat{G}}_{n}$ (see the (2,2) subplots) are: (a) 567, (b) 1, 677, (c) 853, where the size of each support point plotted is proportional to its weight. The run times are: (a) 398 minutes, (b) 472 minutes, (c) 3 minutes. The KKT residuals are: (a) 7.6 × 10^−3^, (b) 3.2 × 10^−6^, (c) 3.2 × 10^−6^. With denser grid points we are able to obtain sharper denoised estimates that reveal finer details of the CMD.

We first consider the astronomy data Gaia-TGAS (Brown et al., 2016) that has been studied in Anderson et al. (2018), where the extreme deconvolution algorithm (Bovy et al., 2011) was used to estimate the true parallax and photometry of every star. This data set contains n=1,363,432 observations ${\left\{Y_{i}\right\}}_{i=1}^{n}\subset R^{2}$, which can be modeled as a Gaussian location mixture with d=2 where $Y_{i}$ is assumed to have density $f_{G^{*},\Sigma_{i}}$ with a known diagonal covariance matrix $\Sigma_{i}$; see (2). We plot the raw data ${\left\{Y_{i}\right\}}_{i=1}^{n}$, the empirical Bayes estimates ${\left\{{\hat{\theta }}_{i}\right\}}_{i=1}^{n}$, the initial grid, and the estimated prior ${\hat{G}}_{n}$ in Figure 5. With such a large $n\approx 10^{6}$, we found that mixsqp can only handle this problem with small m, up to several hundreds. Thus, in Figure 5(a), we use 30 × 30 grid points (i.e., m=900) when solving it by mixsqp. In contrast, we display in Figure 5(b) the solution obtained from our ALM with 100 × 100 grid points. We can see from the empirical Bayes estimates given by mixsqp in Figure 5(a) that the 30 × 30 grid points are not fine enough to denoise this data properly. In contrast, the empirical Bayes estimates obtained by ALM in Figure 5(b) show more shrinkage overall. One can easily see the benefits of working with a large m here — with denser grid points we are able to obtain sharper denoised estimates that reveal finer details of the CMD^9^. Note that REBayes for the Gaia-TGAS data with $n\approx 10^{6}$ and $m=10^{4}$ takes an excessively long time. To demonstrate the performance of REBayes, we conducted an experiment using a subsample of the original data with a size of n=100,000 and a selection of $m=10^{4}$ grid points. Our ALM took approximately 3 minutes, resulting in an empirical Bayes plot in Figure 5(c). On the other hand, applying REBayes to the same subsampled data took approximately 80 minutes. The resulting empirical Bayes plot produced by REBayes was indistinguishable from that of ALM (given in the (1,2) subplot of Figure 5(c)). The residuals (defined in (42)) of the solution x returned by ALM and REBayes are 3.2 × 10^−6^ and 8.4 × 10^−5^, respectively. We also observed that the solution y of problem (P) returned by ALM and REBayes are nearly the same (the difference, measured by the ${\left\|\cdot \right\|}_{2}$ norm, is 8.2 × 10^−6^).

#### Data set 2 (APOGEE).

Our second real data example is taken from the Apache Point Observatory Galactic Evolution Experiment survey (APOGEE) (Majewski et al., 2017). Following the pre-processing in Ratcliffe et al. (2020), the data set contains n=27,135 observations in $R^{19}$.

We first analyze d=2 features picked from the 19 dimensions. For d=2 we use m=100×100 equally spaced grid points inside the minimum axis-aligned bounding box of the raw data (i.e., the smallest rectangle that contains all the data points), that is known to contain all the support points of ${\hat{G}}_{n}$ (see Soloff et al. (2021)).

**Figure 6:**
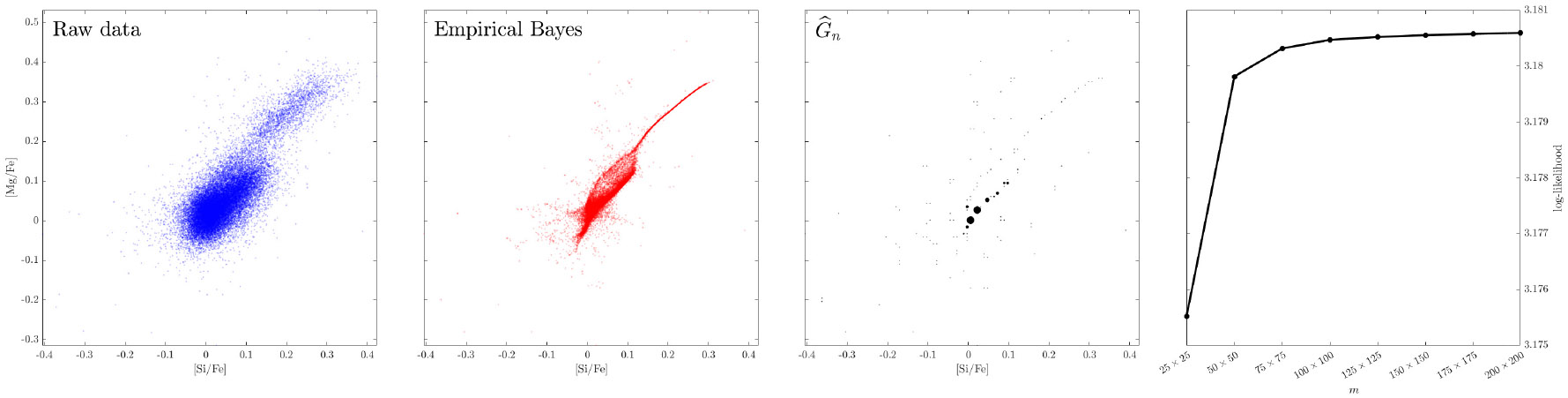
Results for the d=2 dimensional APOGEE data in the [Mg/Fe]-[Si/Fe] plane with $m=100^{2}$ grid points. The empirical Bayes estimates (2nd plot from left) show strong association and a manifold-like structure in the upper right region, and the fitted ${\hat{G}}_{n}$ is very sparse (3rd plot). The rightmost plot gives log-likelihood value against the number of grid points.

**Figure 8:**
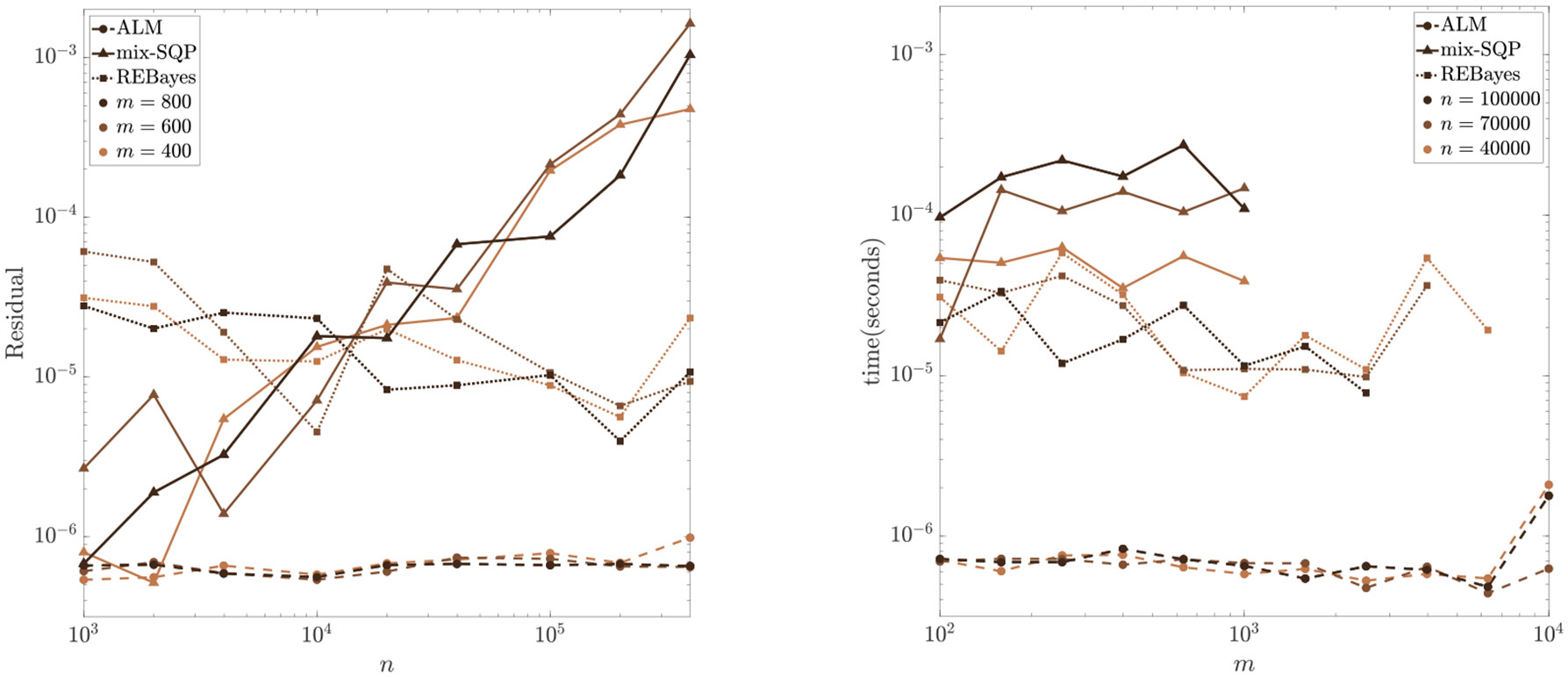
KKT residual (defined in (42)) of the ALM, mixsqp, and REBayes with changing n and m for Example 2 (averaged over 10 replications).

**Figure 9:**
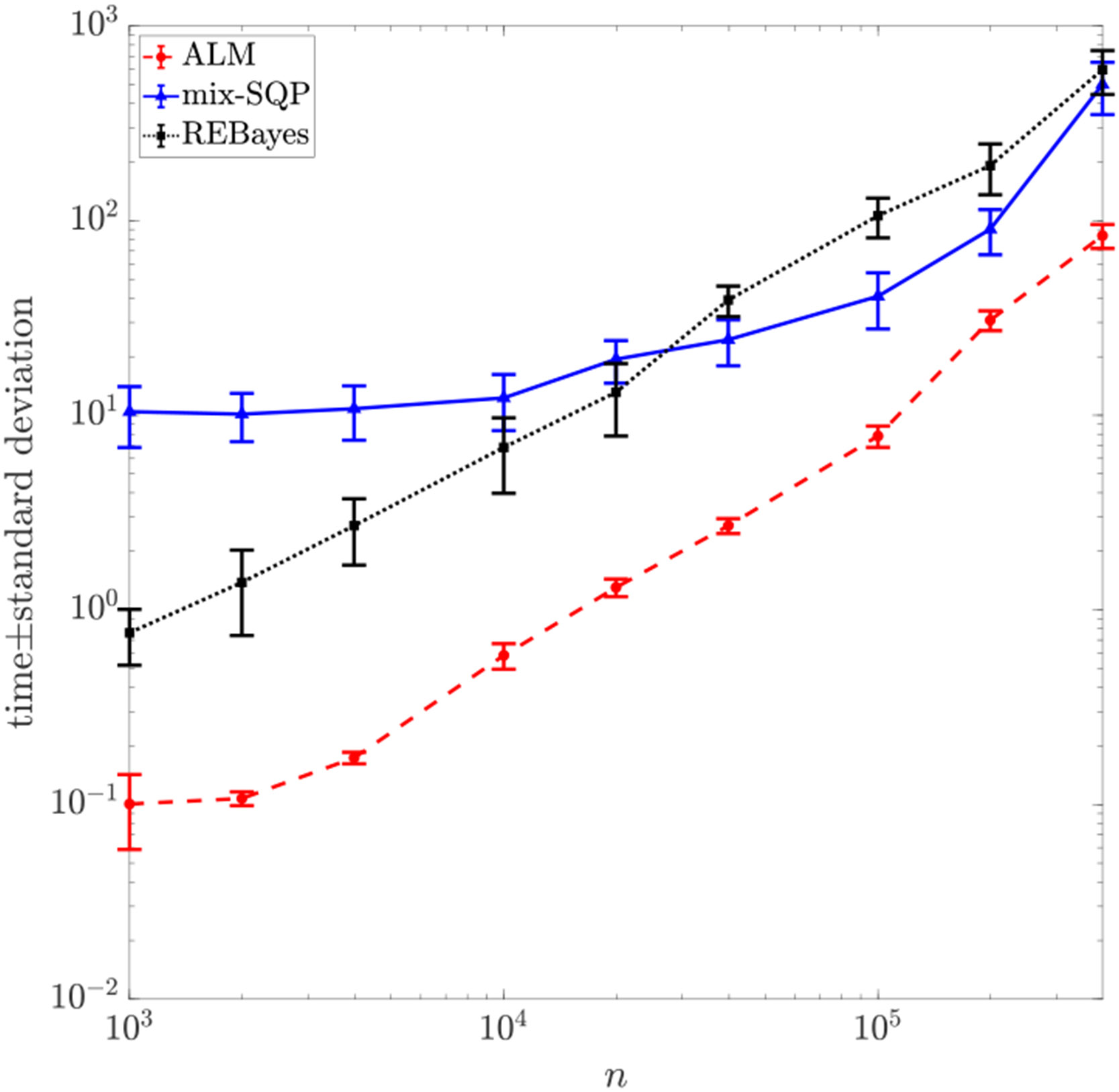
Mean and standard deviation of computational time (in seconds, in the log scale) of the ALM, mixsqp, and REBayes with changing n and m=400 for Example 2 (averaged over 10 replications).

**Figure 10:**
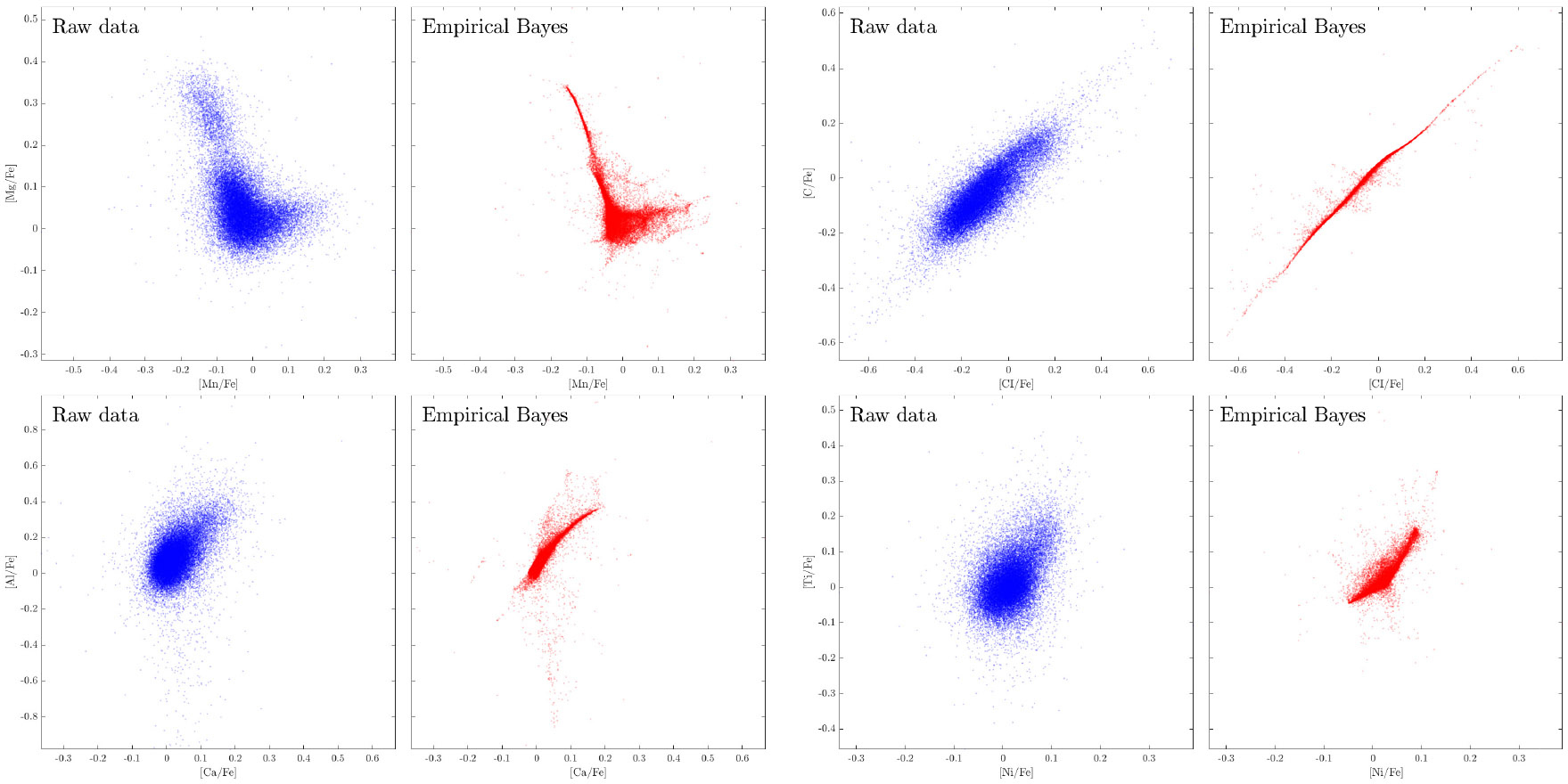
Results from 4 abundance-abundance data sets (with d=2) from the APOGEE survey. Observe that the denoised estimates reveal interesting structure not visible in the raw data plots.

We first illustrate the performance of our ALM on the 2-dimensional plane [Mg/Fe]-[Si/Fe]. The first three plots (from the left) in Figure 6 show the raw data ${\left\{Y_{i}\right\}}_{i=1}^{n}\subset R^{2}$ for n=27,135, 135, the empirical Bayes estimates ${\left\{{\hat{\theta }}_{i}\right\}}_{i=1}^{n}$, and the estimated prior ${\hat{G}}_{n}$. In order to get a sense of how dense the grid points should be to obtain a good approximation of (3) for this data set, we plot the log-likelihood value $\frac{1}{n}\sum_{i=1}^{n}\log\left(\sum_{j=1}^{m}x_{j}\phi_{\Sigma_{i}}(Y_{i}-\mu_{j})\right)$ against the number of grid points $m\in \{25^{2},50^{2},75^{2},100^{2},125^{2},150^{2},175^{2},200^{2}\}$; see the rightmost plot of Figure 6. We see that the objective value improves a lot as the number of grid points increases from 25 × 25 and attains a plateau near 100 × 100. This justifies our choice of taking a set of 100 × 100 grid points for denoising this data set. We provide additional two-dimensional plots along with their denoised empirical Bayes estimates (obtained from a choice of 100 × 100 grid points) in Figure 10 in Appendix C.3. In all the examples we see that the denoised estimates reveal interesting structure not visible in the raw data scatter plots.

To compare the performance of the ALM and REBayes, we select 5 pairs of features from the 19 dimensions (plotted in Figures 6 and 10) and run both algorithms. Table 4 shows that for all instances the ALM is faster than REBayes and the solutions returned by the ALM are more accurate. For this real data set with n=27,135 and m=10,000, mixsqp is not applicable. We remind the reader that we have not incorporated a low-rank approximation of the matrix L here since it does not work well for multivariate data as mentioned in the Introduction; see Figure 2. Therefore, the second-order sparsity in the generalized Hessian mostly contributes to the efficiency of our ALM.

## 5. Conclusion and discussion

In this paper we solve the Lagrangian dual of the optimization problem (6) using a semismooth Newton based augmented Lagrangian method. This approach is highly scalable (e.g., we can solve problems with $n\approx 10^{6}$ and $m\approx 10^{4}$) and it exploits the second-order sparsity in the generalized Hessian matrix arising in the ALM subproblem. We believe that this semismooth Newton based ALM approach is a powerful method for solving large scale optimization problems whose solutions are intrinsically structured sparse (i.e., the solution itself or a linear transformation of the solution is sparse). In fact, this algorithmic framework has already been shown to be effective for the Lasso problem and its variants; see e.g., Li et al. (2018a), Li et al. (2018b), Zhang et al. (2020).

In this paper we have focused our attention on fitting the Gaussian location mixture model (1). However, the scope of our approach is much more general. In fact, one could consider the following d-dimensional (d≥1) observation model:

(31) $$Y_{i}|\theta_{i}∼p_{i}(\cdot |\theta_{i}),\;\text{with}\;\theta_{i}\overset{iid}{∼}G^{*},\;\text{for}\;i\in \{1,\ldots ,n\}$$

where ${\left\{p_{i}(\cdot |\cdot )\right\}}_{i=1}^{n}$ is a sequence of known probability densities and ${\left\{\theta_{i}\right\}}_{i=1}^{n}\subset R^{p}\;(p\geq 1)$ is the sequence of i.i.d. (from $G^{*}$) underlying latent parameters. The algorithm developed in this paper immediately generalizes to this setting as the NPMLE of $G^{*}$ in (31) can be computed similarly. See Example 2 in Section 4.1 where we illustrate this for a *scale* mixture of centered Gaussian distributions.

**Figure 14:**
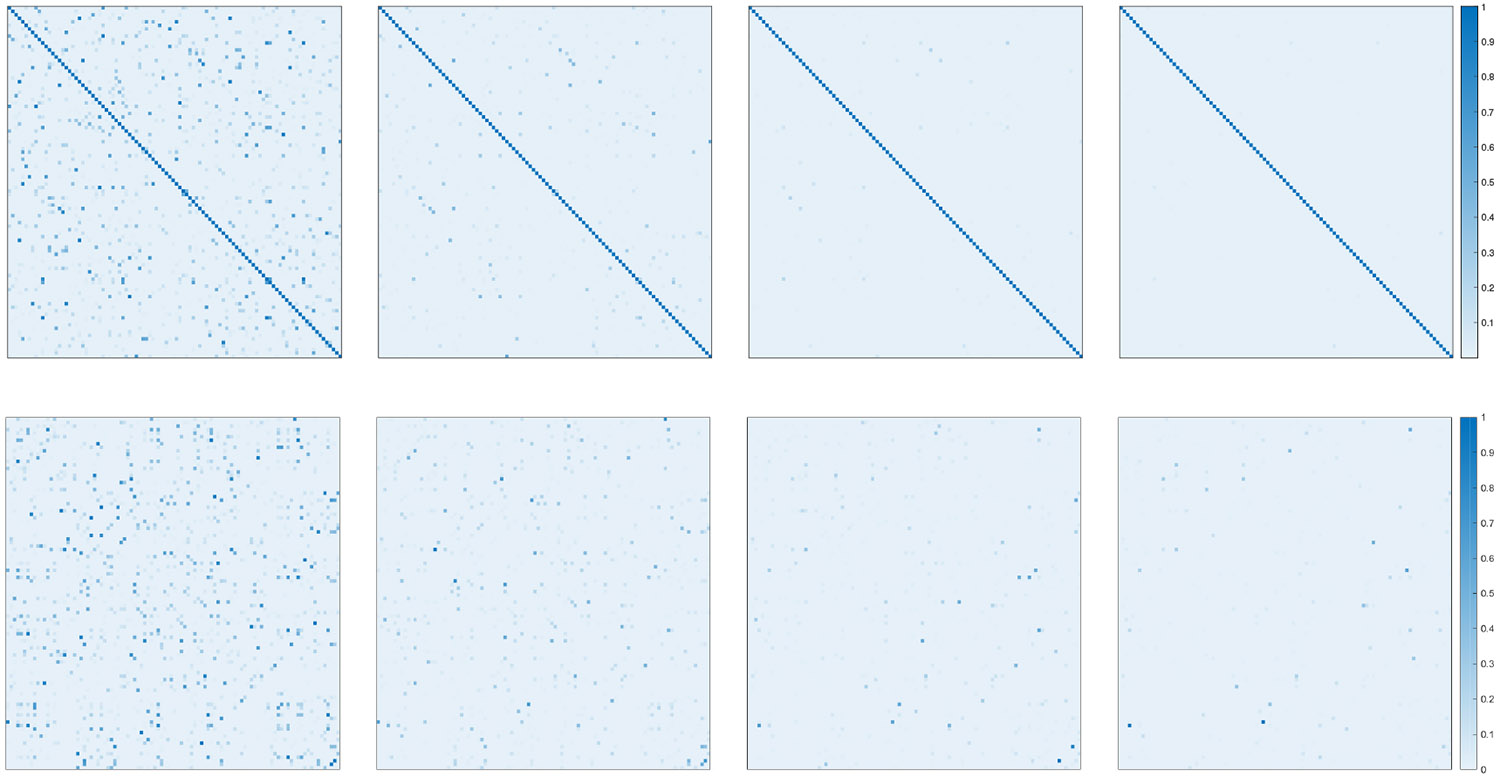
The plots in the first row show the heatmap of the sub-matrix $L_{𝒥,𝒥}$, obtained from data from Example 3(a), as d varies in {3, 6, 9, 12} (from left to right). Here 𝒥 is subsampled randomly from {1,2,…,n} with ∣𝒥∣=100. The plots in the first row show that the matrix L is approaching the identity matrix as d increases. The plots in the second row are obtained after enforcing $L_{ii}=0$ for all i.

The effectiveness of our ALM in estimating ${\hat{G}}_{n}$ and the ${\hat{\theta }}_{i}$’s (as illustrated via simulations and theory) shows the power and scope of nonparametric empirical Bayes as a methodology in multivariate problems. However, when d is large (e.g., d≥10), the NPMLE in (3) can overfit the data; see Appendix C.4 and Appendix C.5 for a detailed numerical study of this phenomenon and plausible explanations (see e.g., Figure 14). This leaves open the study of regularization methods for estimating the unknown $G^{*}$ when d is large. We expect this to be a fruitful direction of future research.

We next discuss on the potential of applying stochastic methods for solving problem (6). Although the stochastic projected gradient method can theoretically be applied, its effectiveness diminishes as the value of m increases. This is primarily due to the computational burden associated with computing the gradient of the objective function in (6) for a single i, not to mention for a batch of i’s. The gradient computation involves evaluating $\sum_{j=1}^{m}L_{ij}x_{j}$, making it computationally expensive. The stochastic projected gradient method typically needs thousands of iterations to achieve a reasonable solution, thus necessitating a large number of expensive high-dimensional matrix-vector multiplications. In contrast, our semismooth Newton based augmented Lagrangian method requires significantly fewer iterations, resulting in a reduced number of gradient evaluations. Moreover, the generalized Hessians in our approach often exhibit sparsity and, in some cases, are computationally more efficient to compute than gradients.

## Figures and Tables

**Table 1:** Computational cost of ALM, interior point method (IPM), and mixsqp for evaluating the gradient and Hessian. The term “*full* L” represents the cost of plain evaluation of the gradient and Hessian, while the term “*rank* k *approx. of* L” lists the reduced cost with the low-rank approximation of L as in (21). Here, n denotes the number of data points, m denotes the number of grid points, s represents the second-order sparsity (19), k denotes the numerical rank of L (21).

	gradient	Hessian
IPM	O(nm)	$O(n^{2}m)$
ALM - full L	O(nm)	O(nsmin(n,s))
ALM - rank k approx. of L	$O((n+m)k+\text{min}{\left(n,m\right)}^{2})$	$O(n\;\text{min}{\left(k,s\right)}^{2})$
mixsqp - full L	O(nm)	$O(nm^{2})$
mixsqp - rank k approx. of L	$O((n+m)k+\text{min}{\left(n,m\right)}^{2})$	$O((n+m)k^{2}+m^{2}k)$

**Table 2:** Comparison between ALM, mixsqp, and REBayes for Example 1 with n=1,000 and m=500 (averaged over 10 replications). The column “Relative objective value” is in terms of the negative log-likelihood value $l=-\frac{1}{n}\sum_{i=1}^{n}\log(\sum_{j=1}^{m}L_{ij}x_{j})$. It represents $\frac{l-l^{*}}{1+|l^{*}|}$, where $l^{*}$ is the smallest value among the three methods and l is the value for a particular method.

τ	ν	Time (in sec)			Residual			Relative objective value		
		
		ALM	mixsqp	REBayes	ALM	mixsqp	REBayes	ALM	mixsqp	REBayes
5	3	0.1	20.6	0.7	4.2e-07	3.4e-06	2.6e-05	6.1e-09	1.2e-07	0
	4	0.1	24.0	0.6	3.8e-07	2.2e-05	2.8e-05	4.8e-09	7.9e-07	0
	5	0.1	25.0	0.8	6.2e-07	1.5e-05	1.5e-05	1.9e-08	1.2e-06	0
	7	0.1	13.0	0.7	4.9e-07	3.3e-03	6.0e-05	1.8e-08	5.5e-06	0
50	3	0.1	21.8	0.6	5.3e-07	6.7e-05	1.6e-05	1.6e-08	1.3e-06	0
	4	0.1	21.7	0.6	4.1e-07	1.8e-04	1.8e-05	1.6e-08	5.1e-06	0
	5	0.1	21.4	0.5	6.2e-07	2.5e-04	1.2e-05	1.2e-08	2.5e-06	0
	7	0.1	17.6	0.7	4.5e-07	3.4e-05	1.6e-05	0	1.9e-06	6.8e-10
500	3	0.1	20.2	0.5	6.2e-07	2.5e-05	2.8e-05	0	1.1e-06	6.3e-10
	4	0.1	20.2	0.5	4.9e-07	3.4e-03	2.5e-05	5.9e-10	6.2e-06	0
	5	0.1	21.0	0.6	4.1e-07	1.6e-05	3.1e-05	0	1.2e-06	9.1e-09
	7	0.1	17.3	0.6	4.6e-07	4.8e-05	3.5e-05	0	1.7e-06	4.1e-09

**Table 3:** Comparison between ALM and REBayes for Example 1 with n=10,000 and m=5,000 (averaged over 10 replications).

τ	ν	Time (in sec)		Residual		Relative objective value	
		
		ALM	REBayes	ALM	REBayes	ALM	REBayes
50	3	3.9	107.8	5.0e-07	4.6e-05	4.1e-09	0
	4	3.8	99.2	6.7e-07	4.1e-05	4.5e-09	0
	5	3.7	103.6	4.8e-07	2.2e-05	1.3e-08	0
	7	3.7	115.8	6.0e-07	7.7e-05	1.6e-08	0
500	3	3.7	112.9	6.0e-07	1.7e-04	1.4e-08	0
	4	3.8	103.6	5.6e-07	1.4e-04	8.0e-09	0
	5	4.1	117.2	5.3e-07	9.2e-05	6.5e-09	0
	7	4.0	132.8	6.1e-07	4.1e-05	6.9e-09	0
5000	3	3.4	90.6	7.0e-07	3.3e-05	1.7e-08	0
	4	3.4	89.4	7.0e-07	3.1e-04	0	2.5e-10
	5	3.6	105.5	7.4e-07	3.5e-05	1.5e-08	0
	7	3.4	183.8	7.3e-07	4.1e-05	0	1.6e-09

**Table 4:** Numerical performance of ALM and REBayes for 5 abundance-abundance data sets (with d=2) from the APOGEE survey.

Plane	Time (in sec)		Residual	
	
	ALM	REBayes	ALM	REBayes
[Mg/Fe]-[Si/Fe]	31.9	454.9	1.0e-06	5.9e-05
[Mg/Fe]-[Mn/Fe]	24.4	321.9	5.2e-07	7.0e-05
[C/Fe]-[CI/Fe]	22.7	665.7	2.9e-07	9.3e-05
[Al/Fe]-[Ca/Fe]	23.5	436.8	1.0e-06	1.1e-04
[Ti/Fe]-[Ni/Fe]	19.9	1150.1	6.9e-07	1.2e-04

**Figure 7:**
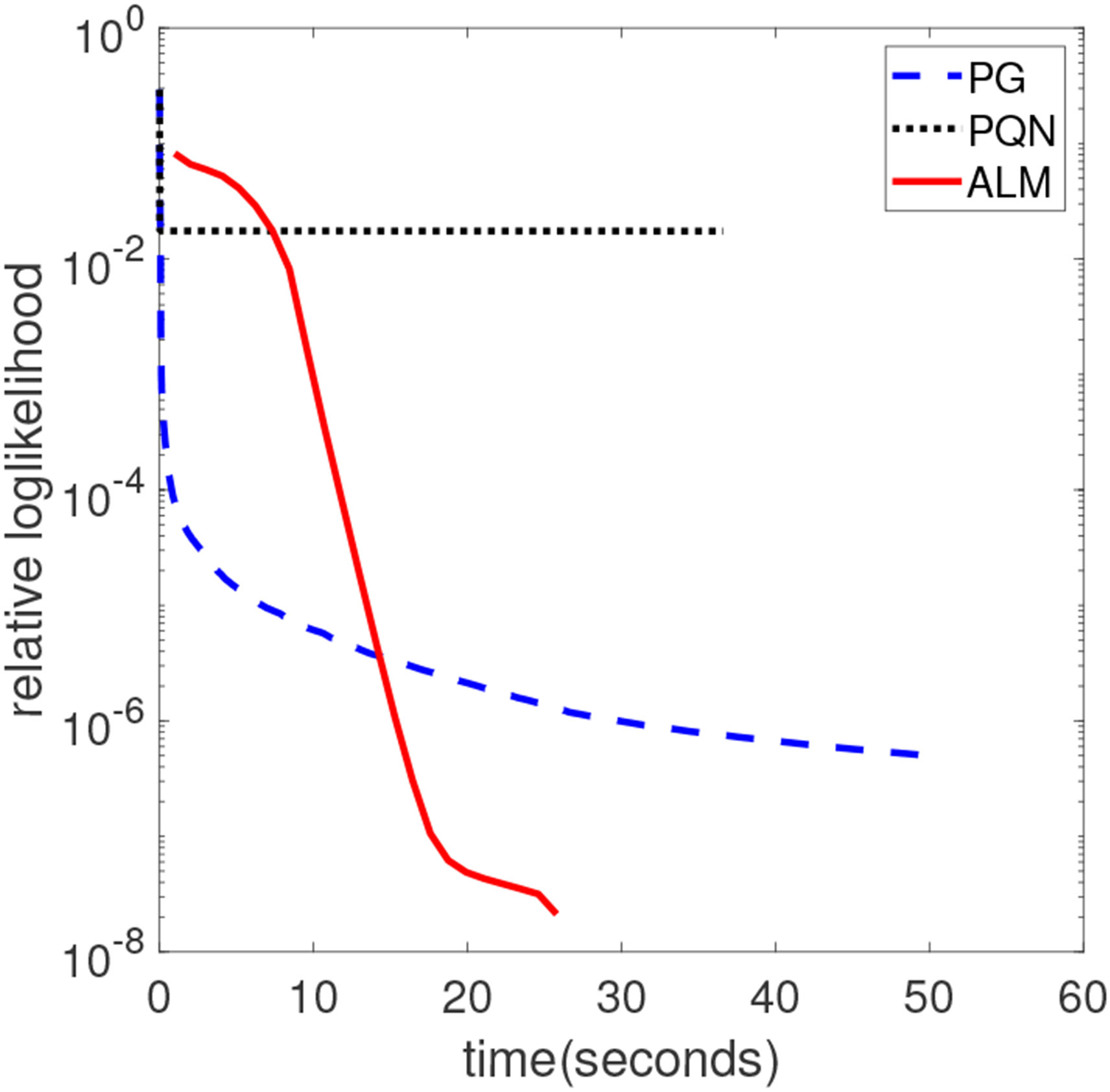
Relative loglikelihood against time on Example 2 with n=5000, m=1000.

**Figure 11:**
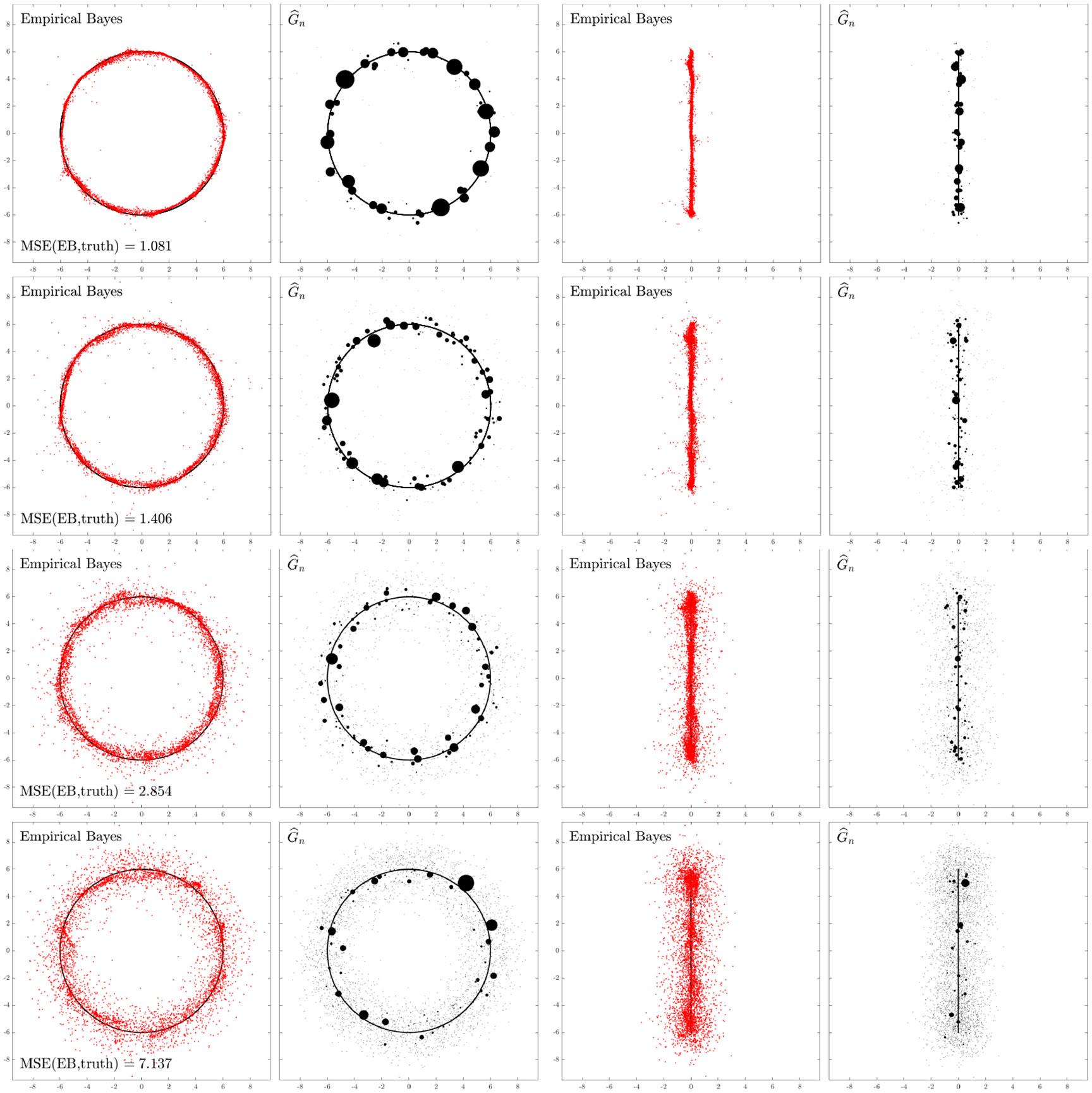
Plots of the projections of the empirical Bayes estimates (in red), the true $G^{*}$ (in black), and ${\hat{G}}_{n}$ (in black dots) obtained from our ALM onto 1-2 plane (see columns 1 and 2) and 2-3 plane (see columns 3 and 4) for data obtained from Example 3(a). The four rows correspond to d=3, 6, 9 and 12 (from top to bottom). Here we take n=m=5,000 and $\mu_{i}=Y_{i}$ for all i=1,…,m. Observe that the quality of the empirical Bayes estimates deteriorates as d increases.

**Figure 12:**
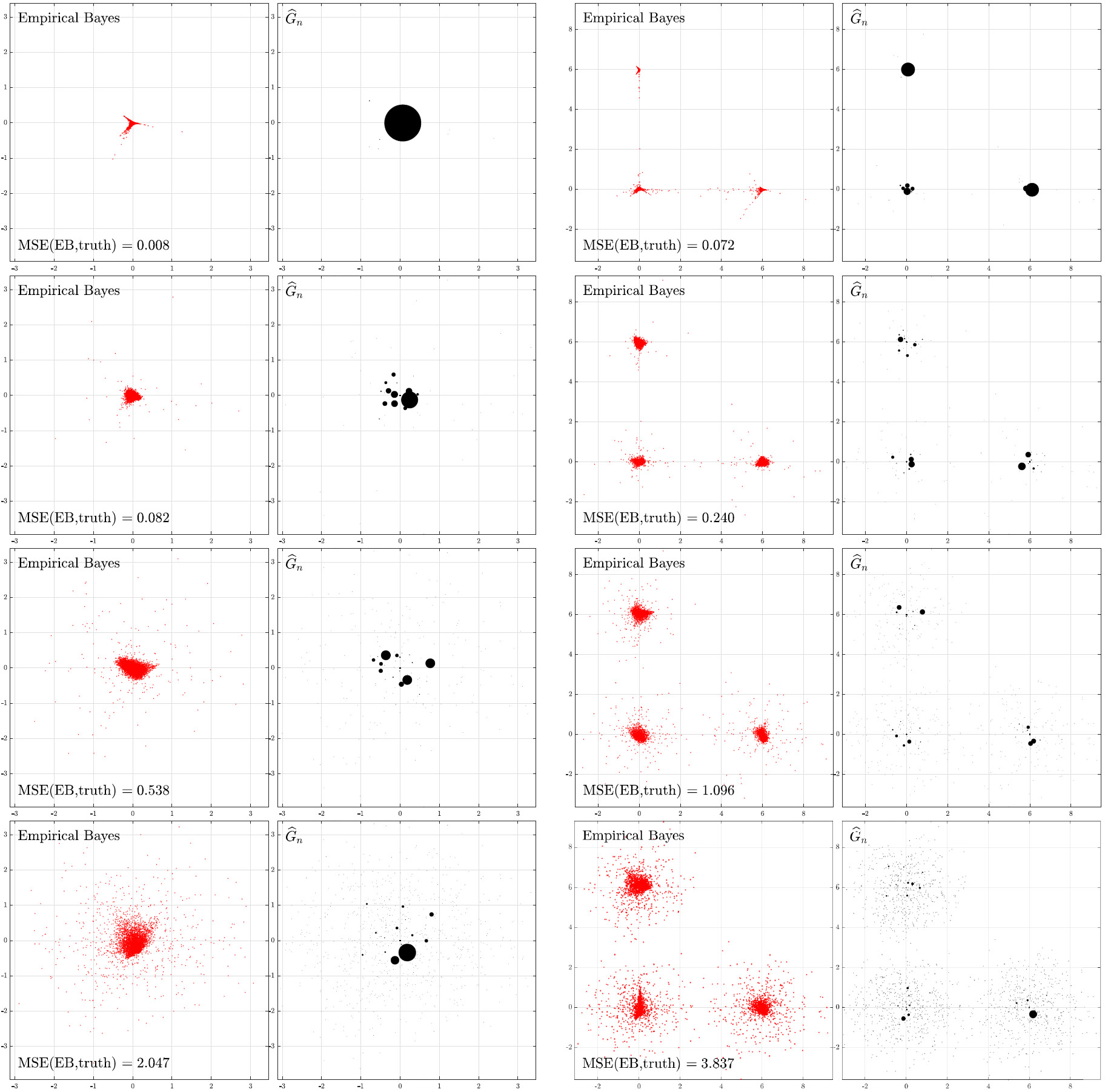
Plots of the projections of the empirical Bayes estimates (in red) and ${\hat{G}}_{n}$ (in black dots) obtained from our ALM for data obtained from Example 3(c) (see columns 1 and 2) and Example 3(b) (see columns 3 and 4). The four rows correspond to d=3, 6, 9 and 12 (from top to bottom). Here we take n=m=5,000 and $\mu_{i}=Y_{i}$ for all j=1,…,n. Observe that the quality of the empirical Bayes estimates deteriorates as d increases.

**Figure 13:**
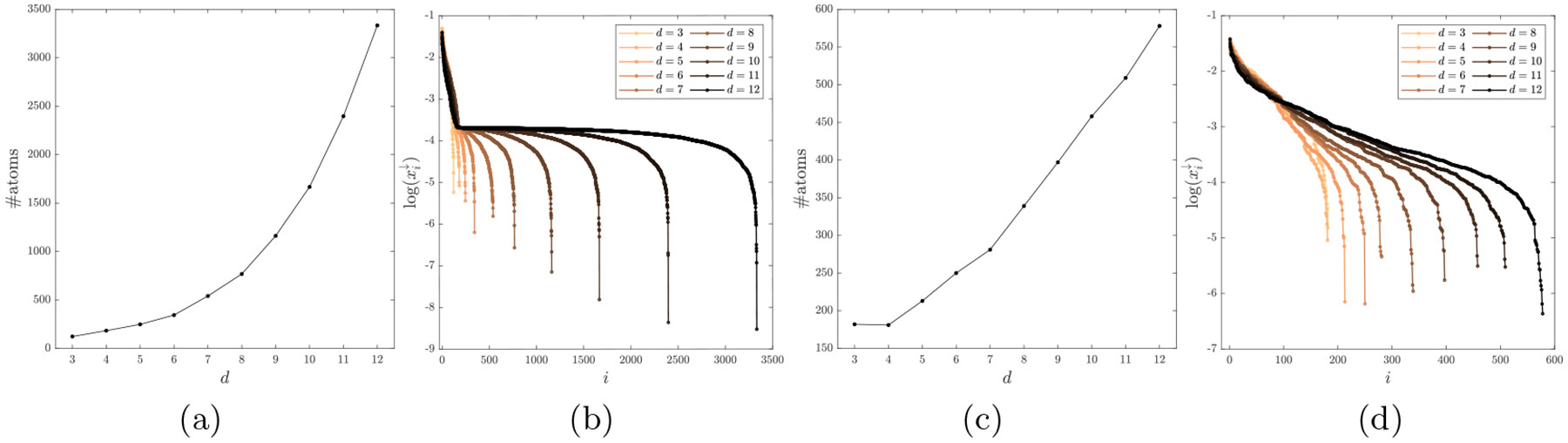
(a) The number of atoms of the estimated ${\hat{G}}_{n}$ (obtained via our ALM) against the dimension d; (b) plot of $\log(x_{i}^{↓})$ for different d, where $x^{↓}$ consists of the sorted elements of the vector x in descending order; (c)-(d) depict similar plots when we incorporate $L_{ii}=0$, for all i, in our ALM. All the plots here are obtained from one run from Example 3(a) with n=m=5,000. Observe that there are more atoms with nonzero weights as d grows.

**Figure 15:**
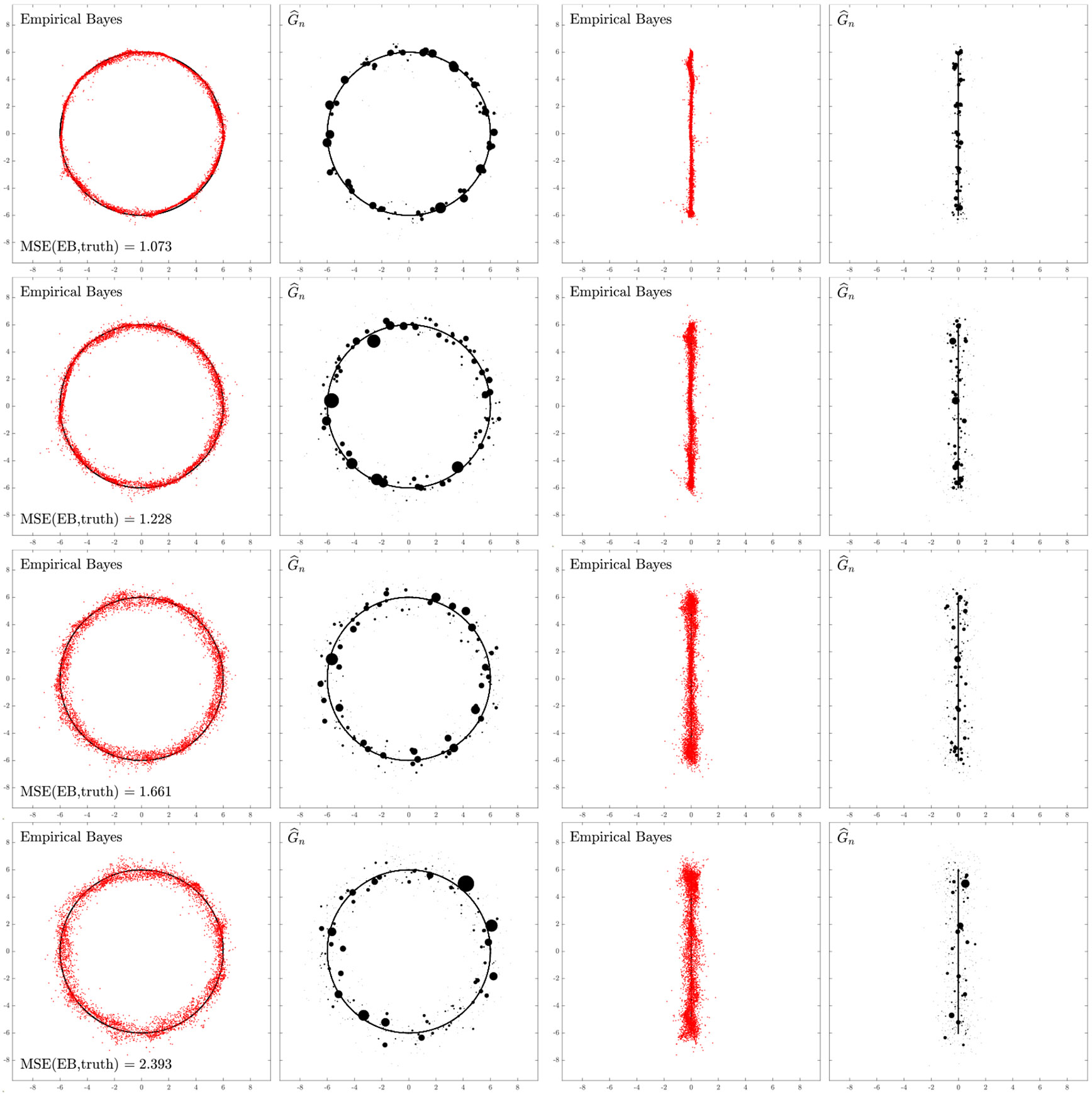
Plots of the projections of the empirical Bayes estimates (in red), the true $G^{*}$ (in black), and ${\hat{G}}_{n}$ (in black dots) obtained from our ALM onto 1-2 plane (see columns 1 and 2) and 2-3 plane (see columns 3 and 4) for data obtained from Example 3(a) where following (44) we enforce $L_{ii}=0$, for all i=1,…,n. The four rows correspond to d=3, 6, 9 and 12 (from top to bottom). Here we take n=m=5,000 and $\mu_{i}=Y_{i}$ for all i=1,…,n.

## Footnotes

1 In particular, we found that the REBayes solver (Koenker and Gu, 2017) reports failure for a synthetic data set with $m=10^{4}$ and $n=7\times 10^{4}$; see Figure 4 in Section 4.1 for details.

2
https://github.com/YangjingZhang/Dual-ALM-for-NPMLE

3 In this paper, the term “dual problem” always refers to the “Lagrangian dual problem”.

4
https://docs.mosek.com/modeling-cookbook/expo.html

5 In this case Monge’s problem is equivalent to Kantorovich’s relaxation as the reference distribution ν is absolutely continuous.

6 Strong convexity here refers to $\psi (z)\geq \psi (x)+\nabla \psi {\left(x\right)}^{T}(z-x)+\frac{\lambda }{2}{\left\|x-z\right\|}^{2}$, for all x, $z\in R^{d}$.

7 By L-smoothness we mean: $\psi (z)\leq \psi (x)+\nabla \psi {\left(x\right)}^{T}(z-x)+\frac{L}{2}{\left\|x-z\right\|}^{2}$, for all x, $z\in R^{d}$.

8 The source code is available at https://github.com/stephenslab/mixsqp.

9 In particular, we point out that 128 GB of RAM was not enough for solving the ALM problem shown in Figure 5(b), since the matrix $L\in R^{1,363,432\times 10,000}$ alone consumes approximately 109 GB of storage. The results in Figure 5(b) were obtained in Matlab (version 9.5) on a Windows workstation (32-core, Intel Xeon Gold 6130 CPU @ 2.10 GHz (2 processors), **256 GB of RAM**) in approximately 472 minutes.

## Appendix A. Preliminaries

### Appendix A.1 Some concepts from convex analysis and optimization

We first introduce some basic notions from convex analysis, including the concept of Moreau-Yosida regularization of a proper closed convex function. A convex function $f\;:R^{n}\to [-\infty ,\infty ]$ is said to be *proper* if f(x)<+∞ for at least one x and f(x)>−∞ for every x. The convex function f is said to be *closed* if {x∣f(x)≤α} is closed for every α∈R. Let $f\;:R^{n}\to (-\infty ,+\infty ]$ be a proper closed convex function. Parametrized by a scalar σ>0, the *Moreau-Yosida regularization* of f (also called the *Moreau envelope* of f) is defined as

(32)
$${ℳ}_{f}^{\sigma }(x)≔\underset{z\in R^{n}}{\text{min}}\left\{f(z)+\frac{\sigma }{2}{\left\|z-x\right\|}_{2}^{2}\right\},\;x\in R^{n};$$

here ${\left\|\cdot \right\|}_{2}$ denotes the usual Euclidean norm. The unique optimal solution of (32) for any given x, denoted as

$${\text{Prox}}_{f}^{\sigma }(x)≔\underset{z\in R^{n}}{\text{argmin}}\left\{f(z)+\frac{\sigma }{2}{\left\|z-x\right\|}_{2}^{2}\right\},$$

is called the *proximal point* of x associated with f. The corresponding function ${\text{Prox}}_{f}^{\sigma }$ is called the *proximal mapping* of f. This regularization is a powerful tool to smooth a possibly nonsmooth convex function such that its gradient can be computed easily based on the proximal mapping of the original function. In fact, one important property is that the Moreau envelope ${ℳ}_{f}^{\sigma }$ is always continuously differentiable (and convex), regardless of whether the original function f is smooth or not, and the function ${ℳ}_{f}^{\sigma }$ has a Lipschitz gradient given by

(33)
$$\nabla {ℳ}_{f}^{\sigma }(x)=\sigma \;[\;x-{\text{Prox}}_{f}^{\sigma }(x)\;]\;,\;x\in R^{n}.$$

Interested readers may consult Rockafellar and Wets (2009, Chapter 1.G) for more properties of the Moreau envelope and the proximal mapping.

Next we introduce the concept of *semismoothness* starting from some basic variational analysis. Let $F\;:\;R^{n}\to R^{m}$ be a vector-valued locally Lipschitz continuous function. It follows from Rademacher’s theorem that F is differentiable almost everywhere. We can thus define the *Clarke generalized Jacobian* of F at any $x\in R^{n}$ as $\partial F(x)≔\mathrm{conv}\{{\text{lim}}_{k\to \infty }JF(x^{k})\;|\;{\left\{x^{k}\right\}}_{k\geq 1}$ is a sequence of differentiable points of F converging to x}, where JF(x) denotes the Jacobian matrix of F; here by conv(S) we mean the convex hull of a given set S. We say F is *semismooth* at $x\in R^{n}$ if F is directionally differentiable at x and for any $V_{h}\in \partial F(x+h)$,

$$F(x+h)-F(x)-V_{h}h=o({\left\|h\right\|}_{2})\;\text{as}\;h\to 0.$$

Detailed properties of semismooth functions can be found in the monograph by Facchinei and Pang (2007).

### Appendix A.2 Introduction to the theory of optimal transport

We present here some notions and results from the theory of optimal transport that is relevant for the paper. This material or slight modifications thereof are accessible from popular monographs and lecture notes on the subject, e.g., Peyré and Cuturi (2019), Villani (2009), Villani (2003), Santambrogio (2015), McCann and Guillen (2011).

**Definition 5 (Push-forward)** Let μ and ν be two Borel probability measures on measurable spaces (𝒳, ${ℬ}_{𝒳}$) and (𝒴, ${ℬ}_{𝒴}$) respectively, and let T be a measurable map from 𝒳 to 𝒴. The map T is said to **push forward** μ to ν, in symbols T#μ=ν, if $T\#\mu (B)\equiv \mu (T^{-1}(B))=\nu (B)$ for all $B\in {ℬ}_{𝒴}$.

**Definition 6 (Monge’s problem)** Let μ and ν be as in the previous definition, and let c:𝒳×𝒴→[0,∞) be a measurable function (“cost function”). The optimal transport problem (Monge’s problem) with μ, ν, and c is given by

(34)
$$\underset{T}{\text{inf}}\int_{𝒳}c(x,T(x))\;d\mu (x)\;\;\;subject\;to\;T\#\mu =\nu .$$

Any minimizer of the above problem is called an optimal transport map.

The following optimization problem is in general a relaxation of the above problem; under certain conditions, both problems are equivalent.

**Definition 7 (Kantorovich’s problem)** Let μ and ν be as in Definition 5, and let c be a cost function as in Definition 6. Let further Π(μ,ν) denote the set of all couplings between μ and ν, i.e., probability measures on 𝒳×𝒴 whose marginals equal to μ and ν. The Kantorovich problem is given by the optimization problem

$$\underset{\gamma \in \Pi (\mu ,\nu )}{\text{inf}}\int_{𝒳}\int_{𝒴}c(x,y)\;d\gamma (x,y).$$

Any minimizer of the above problem is called an optimal transport plan.

For measures μ and ν on $R^{d}$ with finite k-th moments (k≥1), i.e., $\int {\left\|x\right\|}^{k}d\mu (x)<\infty$ and $\int {\left\|x\right\|}^{k}d\nu (x)<\infty$, the k*-Wasserstein distance* between μ and ν is defined via the above Kantorovich problem with cost function $c(x,y)={\left\|x-y\right\|}_{2}^{k}$, i.e.,

$$W_{k}(\mu ,\nu )≔{\left(\underset{\gamma \in \Pi (\mu ,\nu )}{\text{inf}}\int \int {\left\|x-y\right\|}_{2}^{k}\;d\gamma (x,y)\right)}^{1∕k}.$$

A celebrated result due to Brenier characterizes optimal transport maps in the sense of Definition 6 for $𝒳\;=\;𝒴\;=\;R^{d}$ and quadratic cost, i.e., $c(x,y)={\left\|x-y\right\|}_{2}^{2}$ and μ absolutely continuous with respect to the Lebesgue measure. In the sequel, we let $g^{⋆}(x)≔{\text{sup}}_{y\in R^{d}}\{y^{T}x-g(y)\}$ denote the Legendre-Fenchel conjugate of a convex function $g\;:R^{d}\to R\cup \{+\infty \}$.

**Theorem 8 (Brenier’s theorem)** Suppose that μ and ν are Borel probability measures on $R^{d}$ with finite second moments, and suppose further that μ is absolutely continuous with respect to the Lebesgue measure. Then the optimal transport problem (34) with the quadratic cost, i.e., $c(x,y)={\left\|x-y\right\|}_{2}^{2}$ has a (μ-a.e.) unique minimizer T=∇ψ for a convex function $\psi \;:R^{d}\to R\cup \{+\infty \}$. Furthermore, the optimal transport problem and its Kantorovich relaxation are equivalent in the sense that the optimal coupling in Definition 7 is of the form (id×T)#μ. Moreover, if in addition ν is absolutely continuous, then $\nabla \psi^{⋆}$ is the (ν-a.e.) minimizer of the Monge problem transporting ν to μ, and it holds that $\nabla \psi^{⋆}\;\circ \;\nabla \psi (x)=x$ (μ-a.e.), and $\nabla \psi \;\circ \;\nabla \psi^{⋆}(y)=y$ (ν-a.e.).

## Appendix B. Proofs of main results

### Appendix B.1 Proof of Proposition 2

**Proof** In order to use the general results on the convergence rate of the ALM in Rockafellar (1976, Theorem 5), we prove that the quadratic growth condition of the dual problem in (17) holds. Since each entry of the matrix L is nonnegative and each row of L has at least one nonzero entry, one may obtain from the constraint $\frac{1}{n}L^{T}v\leq 1_{m}$ and the nonnegativity of $\bar{v}$ that ${\left\|\bar{u}\right\|}_{\infty }={\left\|\bar{v}\right\|}_{\infty }<+\infty$. Therefore, we may assume without loss of generality that $c≔{\text{sup}}_{(u,v)\in 𝒩}\{{\left\|u\right\|}_{\infty },{\left\|v\right\|}_{\infty }\}<+\infty$ so that $\nabla^{2}h(u)=\frac{1}{n}\text{Diag}(\frac{1}{u_{i}^{2}})\geq \frac{1}{nc^{2}}I_{n}$ for any (u,v)∈𝒩, where $I_{n}$ is the n×n identity matrix. It can be derived that for any (u,v)∈𝒩 that is feasible to problem (D) of the main paper,

$$h(u)\geq \;h(\bar{u})+\nabla h{\left(\bar{u}\right)}^{T}(u-\bar{u})+\frac{1}{nc^{2}}{\left\|u-\bar{u}\right\|}_{2}^{2}\;\;=\;h(\bar{u})-\sum_{i=1}^{n}\frac{1}{n{\bar{u}}_{i}}(u_{i}-{\bar{u}}_{i})+\frac{1}{nc^{2}}{\left\|u-\bar{u}\right\|}_{2}^{2}\;\;=h(\bar{u})-\frac{1}{n}\sum_{i=1}^{n}(L_{i•}\bar{x})(v_{i}-{\bar{v}}_{i})+\frac{1}{nc^{2}}{\left\|u-\bar{u}\right\|}_{2}^{2}\;\;\geq \;h(\bar{u})+\frac{1}{nc^{2}}{\left\|u-\bar{u}\right\|}_{2}^{2}=h(\bar{u})+\frac{1}{2nc^{2}}({\left\|u-\bar{u}\right\|}_{2}^{2}+{\left\|v-\bar{v}\right\|}_{2}^{2})\;,$$

where the last equality and the last inequality follow from the KKT conditions in (15) of the main paper (from the feasibility of v):

$$L_{i•}\bar{x}={\bar{y}}_{i}=\frac{1}{{\bar{u}}_{i}},\;\frac{1}{n}{\bar{v}}^{T}L\bar{x}=1_{m}^{T}\bar{x},\;\bar{x}\geq 0\;\text{and}\;\frac{1}{n}L^{T}v\leq 1_{m}.$$

Therefore, the quadratic growth condition in (17) of the main paper holds for the dual problem holds with $\kappa ={\left(2nc^{2}\right)}^{-1}$. The asymptotically superlinear convergence rate of the sequence ${\left\{(x^{k},y^{k})\right\}}_{k\geq 1}$ generated by the ALM is now a consequence of Rockafellar (1976, Theorem 5). ■

### Appendix B.2 Proof of Theorem 4

**Proof** For notational simplicity, let $G_{n}$ denote the empirical distribution of ${𝒯}^{*}(Y_{1}),\ldots ,{𝒯}^{*}(Y_{n})$ (note the slight change in notation compared to (24) of our main paper); also, we denote by ‖⋅‖ the usual Euclidean norm (instead of ${\left\|\cdot \right\|}_{2}$). Consider an optimal coupling $\hat{\pi }$ between $\nu_{n}$ and ${\hat{G}}_{n}$ minimizing (28) of the main paper, and let ${\hat{\pi }}_{ij}$ denote the resulting probability mass that is assigned to $Y_{i}$ and ${\hat{a}}_{j}$, for 1≤i≤n, and $1\leq j\leq \hat{k}$. Define further $\pi_{j}(Y_{i})={\hat{\pi }}_{ij}n$, for 1≤i≤n, and $1\leq j\leq \hat{k}$. Accordingly, we have ${\hat{\alpha }}_{j}=\frac{1}{n}\sum_{i=1}^{n}\pi_{j}(Y_{i})=\int \pi_{j}(y)\;d\nu_{n}(y)$, for $1\leq j\leq \hat{k}$. Let $\psi^{⋆}$ denote the Legendre-Fenchel conjugate of ψ (recall that ${𝒯}^{*}=\nabla \psi$ with $\psi \;:R^{d}\to R$ being a convex function). We first bound $\int \psi^{⋆}(\theta )\;d{\hat{G}}_{n}(\theta )-\int \psi^{⋆}(\theta )\;dG_{n}(\theta )$ as

(35)
$$\;\sum_{j=1}^{\hat{k}}{\hat{\alpha }}_{j}\psi^{⋆}({\hat{a}}_{j})-\int \psi^{⋆}(\theta )\;dG_{n}(\theta )\;=\int \sum_{j=1}^{\hat{k}}\pi_{j}(y)\psi^{⋆}({\hat{a}}_{j})\;d\nu_{n}(y)-\int \psi^{⋆}({𝒯}^{*}(y))\;d\nu_{n}(y)\;\geq \int \psi^{⋆}\left(\sum_{j=1}^{\hat{k}}\pi_{j}(y){\hat{a}}_{j}\right)d\nu_{n}(y)-\int \psi^{⋆}({𝒯}^{*}(y))\;d\nu_{n}(y)\;=\int \psi^{⋆}({\hat{T}}_{n}(y))\;d\nu_{n}(y)-\int \psi^{⋆}({𝒯}^{*}(y))\;d\nu_{n}(y)\;\geq \int \nabla \psi^{⋆}{\left({𝒯}^{*}(y)\right)}^{T}({\hat{T}}_{n}(y)-{𝒯}^{*}(y))\;d\nu_{n}(y)+\frac{1}{2L}\int {\left\|{\hat{T}}_{n}(y)-{𝒯}^{*}(y)\right\|}^{2}\;d\nu_{n}(x),\;=\int x^{T}({\hat{T}}_{n}(y)-{𝒯}^{*}(y))\;d\nu_{n}(y)+\frac{1}{2L}\int {\left\|{\hat{T}}_{n}(y)-{𝒯}^{*}(y)\right\|}^{2}\;d\nu_{n}(y)$$

where the two inequalities follow from the convexity of $\psi^{⋆}$ (by Jensen’s inequality) and the L-smoothness of ψ, which implies $\frac{1}{L}$-strong convexity of its conjugate $\psi^{⋆}$ (see e.g., Hiriart-Urruty and Lemaréchal (1993)); the last equality follows from Brenier’s theorem (Theorem 8 in Appendix A.2) in light of which $\nabla \psi^{⋆}$ is the inverse map of ∇ψ. Moreover, $W_{2}^{2}(\nu_{n},{\hat{G}}_{n})$ can be expressed as

(36)
$$\sum_{i=1}^{n}\sum_{j=1}^{\hat{k}}{\left\|{\hat{a}}_{j}-Y_{i}\right\|}^{2}{\hat{\pi }}_{ij}=\sum_{j=1}^{\hat{k}}{\hat{\alpha }}_{j}{\left\|{\hat{a}}_{j}\right\|}^{2}+\frac{1}{n}\sum_{i=1}^{n}{\left\|Y_{i}\right\|}^{2}-2\sum_{i=1}^{n}\sum_{j=1}^{\hat{k}}{\hat{a}}_{j}^{T}Y_{i}{\hat{\pi }}_{ij}\;\;=\int {\left\|\theta \right\|}^{2}\;d{\hat{G}}_{n}(\theta )+\int {\left\|y\right\|}^{2}\;d\nu_{n}(y)-\frac{2}{n}\sum_{i=1}^{n}Y_{i}^{T}\left(\sum_{j=1}^{\hat{k}}n{\hat{\pi }}_{ij}{\hat{a}}_{j}\right)\;\;=\int {\left\|\theta \right\|}^{2}\;d{\hat{G}}_{n}(\theta )+\int {\left\|y\right\|}^{2}\;d\nu_{n}(y)-2\int y^{T}{\hat{T}}_{n}(y)\;d\nu_{n}(y).$$

Similarly,

(37)
$$W_{2}^{2}(\nu_{n},G_{n})=\int {\left\|\theta \right\|}^{2}\;dG_{n}(\theta )+\int {\left\|y\right\|}^{2}\;d\nu_{n}(y)-2\int y^{T}{𝒯}^{*}(y)\;d\nu_{n}(y)$$

where we note that ${𝒯}^{*}$ is also the optimal transport map from $\nu_{n}$ to $G_{n}$ (as ${𝒯}^{*}\#\nu_{n}=G_{n}$ and ${𝒯}^{*}$ is the gradient of a convex function). Combining (35), (36), (37), we obtain that

(38)
∫‖T^n(y)−𝒯∗(y)‖2dνn(y)≤L[W22(νn,G^n)−W22(νn,Gn)+2∫ψ⋆(θ)d(G^n−Gn)(θ)]+[∫‖θ‖2d(Gn−G^n)(θ)].

Let $\hat{\eta }$ be an optimal coupling between $G_{n}$ and ${\hat{G}}_{n}$, and let $\eta =(\nabla \psi^{⋆},\mathrm{id})\#\hat{\eta }$ be the pushforward (cf. Definition 5) of the coupling $\hat{\eta }$; note that η has the two marginals to $\nabla \psi^{⋆}\#G_{n}=\nu_{n}$ and $\mathrm{id}\#{\hat{G}}_{n}={\hat{G}}_{n}$, where we have used that $\nabla \psi^{⋆}({𝒯}^{*}(Y_{i}))=Y_{i}$, 1≤i≤n, by Brenier’s theorem, with id denoting the identity map.

Accordingly, by the definition of the 2-Wasserstein distance in terms of optimal couplings (cf. Appendix A.2), we obtain that

$$W_{2}^{2}(\nu_{n},{\hat{G}}_{n})\leq \int {\left\|y-\theta \right\|}^{2}\;d\eta (y,\theta )=\int {\left\|\nabla \psi^{⋆}(\zeta )-\theta \right\|}^{2}\;d\hat{\eta }(\zeta ,\theta ).$$

Adding and subtracting ζ inside the norm on the right-hand side and expanding the square, it follows that

(39)
$$W_{2}^{2}(\nu_{n},{\hat{G}}_{n})\leq \int {\left\|\nabla \psi^{⋆}(\zeta )-\zeta \right\|}^{2}\;dG_{n}(\zeta )+\int {\left\|\theta -\zeta \right\|}^{2}\;d\hat{\eta }(\zeta ,\theta )+2\int {\left(\nabla \psi^{⋆}(\zeta )-\zeta \right)}^{T}(\zeta -\theta )\;d\hat{\eta }(\zeta ,\theta )\;\;=W_{2}^{2}(G_{n},\nu_{n})+W_{2}^{2}(G_{n},{\hat{G}}_{n})+2\int {\left(\nabla \psi^{⋆}(\zeta )-\zeta \right)}^{T}(\zeta -\theta )\;d\hat{\eta }(\zeta ,\theta ),$$

where we have used that $\nabla \psi^{⋆}$ is the OT map pushing forward $G_{n}$ to $\nu_{n}$, the definition of the 2-Wasserstein distance in terms of optimal transport and optimal couplings, and the definition of $\hat{\eta }$ as optimal coupling between $G_{n}$ and ${\hat{G}}_{n}$.

In order to bound the rightmost term in the preceding display, we use the fact that the function $\psi^{⋆}$ is (1∕λ)-smooth. This yields

(40)
$$2\int \nabla \psi^{⋆}{\left(\zeta \right)}^{T}(\zeta -\theta )\;d\hat{\eta }(\zeta ,\theta )\leq 2\int \left\{\psi^{⋆}(\zeta )-\psi^{⋆}(\theta )+\frac{1}{2\lambda }{\left\|\zeta -\theta \right\|}^{2}\right\}d\hat{\eta }(\zeta ,\theta )\;\;=2\int \psi^{⋆}(\zeta )\;dG_{n}(\zeta )-2\int \psi^{⋆}(\theta )\;d{\hat{G}}_{n}(\theta )+\frac{1}{\lambda }W_{2}^{2}(G_{n},{\hat{G}}_{n}),$$

where we have used the fact that $\hat{\eta }$ be an optimal coupling between $G_{n}$ and ${\hat{G}}_{n}$. Finally, note that

(41)
$$2\int {\left(-\zeta \right)}^{T}(\zeta -\theta )\;d\hat{\eta }(\zeta ,\theta )=\int \{{\left\|\theta \right\|}^{2}-{\left\|\theta -\zeta \right\|}^{2}-{\left\|\zeta \right\|}^{2}\}\;d\hat{\eta }(\zeta ,\theta )\;\;=\int {\left\|\theta \right\|}^{2}\;d{\hat{G}}_{n}(\theta )-\int {\left\|\zeta \right\|}^{2}\;dG_{n}(\zeta )-W_{2}^{2}(G_{n},{\hat{G}}_{n}).$$

Combining (39), (40), and (41), we obtain that

$$W_{2}^{2}(\nu_{n},{\hat{G}}_{n})\leq W_{2}^{2}(\nu_{n},G_{n})+\frac{1}{\lambda }W_{2}^{2}(G_{n},{\hat{G}}_{n})+2\int \psi^{⋆}(\zeta )\;dG_{n}(\zeta )-2\int \psi^{⋆}(\theta )\;d{\hat{G}}_{n}(\theta )\;\;+\int {\left\|\theta \right\|}^{2}\;d{\hat{G}}_{n}(\theta )-\int {\left\|\zeta \right\|}^{2}\;dG_{n}(\zeta ).$$

Substituting this bound back into (38), we observe that the right-hand side of the display equals $\frac{L}{\lambda }W_{2}^{2}(G_{n},{\hat{G}}_{n})$. The desired result now follows from the above fact in conjunction with the following result: In Soloff et al. (2021, Theorem 10), it is shown that if $G^{*}({\left[-M,M\right]}^{d})=1$, for some M>0, then there is a function n(d,M) such that, for all sample sizes n with n≥n(d,M),

$$W_{2}^{2}(G^{*},{\hat{G}}_{n})\leq C_{d,\sigma }\frac{1}{\log\;n},$$

for a constant $C_{d,\sigma }$, with probability at least $1-\frac{4d}{n^{8}}$.

## Appendix C. Implementation details and additional numerical results

### Appendix C.1 Stopping criteria and implementation details

We first give some details of the stopping conditions for each of the competing methods — our ALM, the mixsqp (Kim et al., 2020) solver, the R package REBayes (Koenker and Gu, 2017). For a given tolerance ε>0, the mixsqp is terminated if

$$\eta_{1}≔\max_{1\leq j\leq m}\left[\frac{1}{n}L_{•j}^{T}(1_{n}⊘Lx)-1\right]\leq \varepsilon ,$$

where $L_{•j}$ represents the j-th column of L and ⊘ denotes the Hadamard division defined as: $x⊘y=(x_{1}∕y_{1},\ldots ,x_{n}∕y_{n})$, for x, $y\in R^{n}$. For REBayes, we adopt its default termination condition with the relative tolerance of the dual gap “rtol” being 10^−6^. We terminate our semismooth Newton based ALM under the following stricter condition

(42)
$$\max(\eta_{1},\eta_{2})\leq \varepsilon ,$$

which additionally involves the KKT residual defined as

$$\eta_{2}≔{\left\|x-\max\left(x+\frac{1}{n}L^{T}(1_{n}⊘Lx)-1_{m},0\right)\right\|}_{2}.$$

In the reported tables and figures, the residual term is computed from $\max(\eta_{1},\eta_{2})$ given in (42). We say a solution is more accurate if its residual is smaller. Throughout our numerical experiments, we set $\varepsilon =10^{-6}$ for our ALM and for mixsqp. In addition, both methods are also terminated if the number of ALM/SQP iterations reaches 100.

In addition, we make the following remarks on the implementation of our ALM:

(a) To further enhance the scalability of the ALM, we borrow a low-rank approximation idea from Kim et al. (2020) for reducing the cost of computing the gradient $\nabla \phi_{k}(\cdot )$ in (11). As shown in Figure 2, the matrix L usually has a lot of singular values close to 0 when d=1 (although this is not the case for d≥2). In our implementations, the ALM constructs a low-rank approximation of L for all experiments only in Section 4.1 where d=1. However, when d≥2, the ALM does not employ a low-rank approximation of L since L is no longer approximately low rank. It is important to note that we do not utilize the low-rank approximation of L when computing the generalized Hessians in our ALM.

(b) The solution of (P) is invariant to scaling each row of L since for any $\alpha_{1},\ldots ,\alpha_{n}>0$,

$$\sum_{i=1}^{n}\log\;{\left(Lx\right)}_{i}=\sum_{i=1}^{n}\log\;(L_{i•}x)=\sum_{i=1}^{n}\log\;(\alpha_{i}L_{i•}x)-\sum_{i=1}^{n}\log\;\alpha_{i},$$

where $L_{i•}$ denotes the i-th row of L. Therefore, we assume without loss of generality that the largest component in each row of L is always 1 by taking $\alpha_{i}=1∕(\max_{1\leq j\leq m}L_{ij})$.

(c) Based on the KKT optimality conditions in (15), we consider the following initial point for our ALM:

$$x^{0}≔\frac{1}{m}1_{m},\;y^{0}≔Lx^{0}=\frac{1}{m}L1_{m},\;v^{0}=u^{0}≔1_{m}⊘y^{0}.$$

We find that this initial point works well for the synthetic data sets tested in Section 4.1. For the more challenging real data sets in Section 4.2, we construct the following initial point to fully take advantage of the second-order sparsity in the generalized Hessian matrix appearing in the ALM subproblem:

(43)
$$x^{0}≔\frac{\sigma_{0}}{2}1_{m},\;y^{0}≔\frac{1}{m}L1_{m},\;u^{0}≔1_{m}⊘y^{0},\;v^{0}≔0_{n}.$$

Recall from Table 1 that the computational cost of finding the generalized Hessian in our ALM is O(nsmin(n,s)), where $s=\{i\;:S_{ii}\neq 0\}$ is the number of nonzero entries in the diagonal matrix S; see (13). The idea behind the above initialization is to make s as small as possible for the first several ALM iterations (and semismooth Newton sub-iterations). Starting from the initial point in (43), we have that for the first ALM subproblem (i.e., $x=x^{0}$), it holds that for any dual variable $v\in R^{n}$,

$$S\in \partial \;\max\left(\frac{1}{n}L^{T}v-1_{m}+\frac{1}{\sigma_{0}}x^{0},0\right)=\partial \;\max\left(\frac{1}{n}L^{T}v-\frac{1}{2}1_{m},0\right).$$

Since we set $v^{0}=0_{n}$, it always holds that $\frac{1}{n}L^{T}v^{0}-\frac{1}{2}1_{m}\leq 0$ and S can be taken as a zero matrix for the first semismooth Newton iteration (within the first ALM subproblem). As the algorithm proceeds, the variable v will deviate from the zero vector gradually, and the number of violated inequalities in $\frac{1}{n}L^{T}v-\frac{1}{2}1_{m}\leq 0$ may increase correspondingly. Hence, the choice of the initial point in (43) would result in a gradually increasing s (from zero) that helps to reduce the computational cost in the early iterations of the algorithm.

(d) Once we obtain an approximate solution x to problem (P) via the ALM, we renormalize $x\;(x\mapsto x∕\sum_{j=1}^{m}x_{j})$ such that its components add up to one.

(e) We next give the adjustment of the positive scalar $\sigma_{k}$ in the ALM framework. It follows from Proposition 2 that a larger $\sigma_{k}$ gives rise to a faster local convergence rate $(\kappa ∕\sqrt{\kappa^{2}+\sigma_{k}^{2}}+\eta_{k}){\left(1-\eta_{k}\right)}^{-1}$. However, when $\sigma_{k}$ is very large, the condition number of the the generalized Hessian matrix in (13) will be large since the diagonal entries of the positive definite diagonal matrix $D^{k}$ will be close to zero. In this case, finding the Newton direction in (14) may need more conjugate gradient steps. Therefore, we shall consider the trade-off between the convergence rate of the ALM and the cost of solving the linear systems in the semismooth Newton method. In our implementation, we set $\sigma_{0}=100$, $\sigma_{k+1}=\sqrt{3}\sigma_{k}$ if $\chi_{k}∕\chi_{k-1}>0.6$, and $\sigma_{k+1}=\sigma_{k}$ otherwise. Here $\chi_{k}≔\max(\max(\frac{1}{n}L^{T}v^{k}-1_{m},0),\left\|u^{k}-v^{k}\right\|∕\|u^{k}\|)$ characterizes the feasibility of (D). Namely, when the improvement on the feasibility of (D) after one iteration is too small, we increase $\sigma_{k}$. Next, the sequence ${\left\{\varepsilon_{k}\right\}}_{k\geq 1}$ (the same for $\left\{\eta_{k}\right\}$) in the stopping criteria satisfying (16) in the main paper is chosen as follows: $\varepsilon_{0}=0.5$, $\varepsilon_{k+1}=\varepsilon_{k}∕\varsigma$. We set ς=1.06 if the k-th subproblem has been solved efficiently within 30 semismooth Newton iterations; otherwise, we set ς=1. Lastly, in the semismooth Newton method (Algorithm 2), we set $\bar{\eta }=0.1$, τ=0.1, $\mu =10^{-4}$, and β=0.5.

(f) The Newton system (14) can be solved by direct solvers, for example, via computing the Cholesky factorization of the coefficient matrix, when the size of the coefficient matrix is moderate (≤ 5, 000). Alternatively, when the dimension of the linear system is large (> 5, 000), we solve it iteratively by conjugate gradient method.

### Appendix C.2 Comparison with first-order methods

First order methods are natural choices for solving large scale problems. In this section, we conduct preliminary experiments to demonstrate the convergence behaviour of first-order methods. We compare our ALM with two first-order methods: a projected gradient method (PG) and a limited-memory projected quasi-Newton method (PQN). The implementation of these methods was based on the MATLAB codes “minConf_SPG.m” and “minConf_PQN.m” developed by Mark Schimidt, which can be obtained from the following link: https://www.cs.ubc.ca/~schmidtm/Software/minConf.html. We conducted experiments for Example 2 in Section 4.1, with n=5000 and m=1000. In Figure 7, we present the relative objective value, which represents the difference between the loglikelihood value at the current iterate and the log-likelihood value at the best solution among the three compared methods, plotted against the running time for the projected gradient method (PG), the limited-memory projected quasi-Newton method (PQN), and our proposed ALM. Figure 7 clearly demonstrates that the two first-order methods exhibit rapid progress during the initial stages of optimization. However, as they approach the solution, their convergence significantly slows down. This behavior suggests that although first-order methods can quickly generate approximate solutions with low accuracy, they usually stagnate after some iterations and cannot make further (substantial) progress. In contrast, our ALM showcases superior performance, consistently making progress throughout the optimization process. In particular, our ALM has fast convergence when the iterates are close to the true solution. These results highlight the advantages of our ALM compared to first-order methods even in the considered large scale setting.

Here we do not conduct a comprehensive comparison between first-order methods (e.g., PG, PQN) and second-order methods (e.g., our proposed ALM). We can still gain the insights that first-order methods exhibit rapid progress during the initial stages of optimization, whereas second-order methods demonstrate rapid convergence when the iterates approach the true solution. Consequently, employing first-order methods as an initial warmstart phase, followed by the subsequent application of second-order methods to achieve highly accurate solutions, could further accelerate the optimization process. In fact, the combination of first-order methods and second-order methods is a widely used technique in the field of optimization; see e.g., Li et al. (2018c), Li et al. (2020, Section 5), Zhang et al. (2021, Section 4.1.2).

### Appendix C.3 Additional numerical results

Additional results on Example 2 in Section 4.1 are given in Figures 8 and 9.

For APOGEE data, we provide additional two-dimensional abundance-abundance plots along with their denoised empirical Bayes estimates (obtained from a choice of 100 × 100 grid points) in Figure 10. In all the examples we see that the denoised estimates reveal interesting structure not visible in the raw data plots. In particular, the empirical Bayes estimates of the data in the [C/Fe] and [CI/Fe] plane (see the top right plots in Figure 10) reveal very strong (almost linear) association between the two variables.

### Appendix C.4 Performance of the ALM when d≥3

In this subsection, we consider multivariate synthetic data. In this multivariate setting the discretization of (3) based on equally spaced grid points in a compact region of $R^{d}$ is no longer feasible since the number of such grid points grow exponentially in the number of dimensions d, e.g., $m=100^{d}$. Therefore, when d≥3 we simply take all observations as grid points, i.e., we take m=n and $\mu_{i}=Y_{i}$ for all i=1,…,n; this approach was advocated by Lashkari and Golland (2007).

We consider the following settings where ${\left\{\theta_{i}\right\}}_{i=1}^{n}\subset R^{d}$ is generated as:

3(a) The first two coordinates of $\theta_{i}\in R^{d}$ are drawn uniformly at random from the circle of radius 6 (centered at $(0,0)\in R^{2}$), and the remaining entries are set to zero;
3(b) Each $\theta_{i}$ is generated independently from the discrete distribution $G^{*}=\frac{1}{3}(\delta_{e_{1}}+\delta_{e_{2}}+\delta_{e_{3}})$, where $e_{1}=(0,\ldots ,0)\in R^{d}$, $e_{2}=(6,0,0,\ldots ,0)\in R^{d}$, and $e_{3}=(0,6,0,\ldots ,0)\in R^{d}$;
3(c) $\theta_{i}=0\in R^{d}$, for all i=1,…,n.

Given the $\theta_{i}$’s, the observed data are generated independently according to $Y_{i}∼𝒩(\theta_{i},I_{d})$, i.e., we consider the homoscedastic setting $\Sigma_{i}\equiv I_{d}$ (for simplicity). We set n=5,000 and the dimension of the problem d is varied within the set {3, 4, … , 12}.

#### Behavior of the empirical Bayes estimates as d increases:

We illustrate the results for data generated from Example 3(a) using our ALM with the grid points chosen as our data points (here n=m=5,000). Figure 11 displays the projected empirical Bayes estimates onto the first two dimensions and the second and third dimensions for d∈{3,6,9,12}. The plots indicate that the quality of the empirical Bayes estimates deteriorates as d increases. This phenomenon is also observed on Examples 3(b,c) (see Figure 12) and is intuitively expected since the task of Gaussian denoising gets more difficult as d grows.

#### Behavior of ${\hat{G}}_{n}$ as d increases:

Additionally, we observe from the plots in Figure 11 that the estimated ${\hat{G}}_{n}$ (obtained via our ALM) has more atoms (support points) as d grows. To further illustrate this phenomenon we plot (for this data example) the number of atoms, i.e., the number of nonzero entries of the solution x to problem (6), against the dimension d in Figure 13(a) and the weights of the atoms in Figure 13(b). In particular, we observe that when d=12 about 3,400 atoms (out of 5,000 grid points) have nonzero mass and most of them (excluding the largest/smallest 200; see Figure 13(b)) have mass approximately 2 × 10^−4^. We claim that the main reason for this behavior of the estimated ${\hat{G}}_{n}$ is that the matrix L in (6) approaches the identity matrix as d increases. This is because the diagonal entries $L_{ii}=\phi_{\Sigma_{i}}(Y_{i}-\mu_{i})=\phi_{\Sigma_{i}}(0)$ dominate the off-diagonal entries $L_{ij}=\phi_{\Sigma_{i}}(Y_{i}-\mu_{j})$ (for i≠j) which are typically much smaller, as most points are far from each other in high dimensions; see Remark 9 for a more detailed explanation of this phenomenon.

#### An effective strategy to mitigate this curse of dimensionality:

When d≥3, we set all the diagonal entries of L to zero, but keep all the off-diagonal entries of L intact. This slight modification of L enhances the performance of the obtained ${\hat{G}}_{n}$ and the resulting empirical Bayes estimates; e.g., when d=12 the mean squared error (MSE) between $\hat{\theta }$ and θ, defined as $\frac{1}{n}\sum_{i=1}^{n}{\left\|{\hat{\theta }}_{i}-\theta_{i}\right\|}_{2}^{2}$, equals 2.393 with this adjustment whereas MSE = 7.137 without this tweak (see the plots in Figure 11 and compare with Figure 15).

**Remark 9** (${\hat{G}}_{n}$ **computed via the ALM as** d **increases)** To explain the behavior of the estimated ${\hat{G}}_{n}$ obtained by the ALM, as d increases, we plot the heatmap of the scaled^10^ matrix L computed from data obtained from Example 3(a) for d=3, 6, 9, 12 in the first row of Figure 14. In the heatmap^11^, the values of the entries in L are represented by colors in each square. We can infer from the first row of Figure 14 that the matrix L is approaching the identity matrix as d increases. This is because the diagonal entries $L_{ii}=\phi_{\Sigma_{i}}(Y_{i}-\mu_{i})=\phi_{\Sigma_{i}}(0)$ dominate the off-diagonal entries $L_{ij}=\phi_{\Sigma_{i}}(Y_{i}-\mu_{j})$ (for i≠j) which are typically much smaller, as most points are far from each other in high dimensions. Note that when $L=I_{m}$, we know that $x=\frac{1}{m}1_{m}$ is the solution to (6) and thus the number of support points of ${\hat{G}}_{n}$ should be n=m. This explains why the number of atoms of ${\hat{G}}_{n}$ is increasing with d, and each (non-zero) weight is approaching a fixed value; cf. Figure 13(b).

### Appendix C.5 A strategy to mitigate the curse of dimensionality of the ALM

We introduce the following strategy to slightly modify the matrix L for the computation of the ALM via (6) when d≥3, and the support points are taken to be exactly the data points, i.e., $\mu_{i}=Y_{i}$ for i=1,…,n. This modification enhances the performance of the obtained ${\hat{G}}_{n}$ and the resulting empirical Bayes estimates obtained from the ALM. The strategy is to set all the diagonal entries of L to zero, but keep all the off-diagonal entries of L intact. Namely, we redefine L as:

(44)
Lij={ϕΣi(Yi−μj),ifi≠j,0,ifi=j.}

This adjustment mitigates the domination of the diagonal entries over the off-diagonal entries in L when d is large (as can be seen from the plots in the second row of Figure 14). We found from our extensive simulation experiments that this simple modification can generally improve the denoising results, especially when d is moderately large (e.g., when d varies between 5 and 15); see Figure 15 and compare with Figure 11.

This strategy also yields a ${\hat{G}}_{n}$ with less support points that provides a better estimator of $G^{*}$; see Figure 13(c)-(d) and Figure 15. We can see from Figure 13(c) that most of the grid points have zero mass (e.g., only about 600 of the 5,000 grid points have nonzero mass when d=12). By comparing Figure 11 and Figure 15, we can see that for small dimensions (e.g., d=3) this tweak does not have much effect, whereas for moderate dimensions (e.g., d=12) the effect can be substantial.

## Appendix D. Alternative approach to developing ALM

There is an alternative approach to developing an ALM for solving (D). We can introduces nonnegative slack variables to handle the inequality constraint and then apply the “conventional” ALM used for equality-constrained problems; see e.g., Nocedal and Wright (2006, Chapter 17.3). Next we show that the alternative approach to developing ALM is equivalent to our proposed ALM.

We could introduce nonnegative slack variable s≥0 for the inequality constraint as follows:

(D’)
$$\underset{u,v,s}{\text{minimize}}\;h(u)+\delta_{\geq 0}(s)\;\;\;\text{subject to}\;\frac{1}{n}L^{T}v+s=1_{m},\;\;u-v=0.$$

The “conventional” augmented Lagrangian function for (D’) can be written as follows: for σ>0, $x\in R^{m}$, $y\in R^{n}$,

(45)
$${\tilde{L}}_{\sigma }(u,v,s;x,y)≔h(u)+\delta_{\geq 0}(s)+y^{T}(u-v)+\frac{\sigma }{2}{\left\|u-v\right\|}_{2}^{2}\;\;+x^{T}\left(\frac{1}{n}L^{T}v+s-1_{m}\right)+\frac{\sigma }{2}{\left\|\frac{1}{n}L^{T}v+s-1_{m}\right\|}_{2}^{2}.$$

Here we leave the nonnegativity of the slack variable s in the objective function via an indicator function $\delta_{\geq 0}(s)$ such that $\delta_{\geq 0}(s)=0$ if s≥0 and $\delta_{\geq 0}(s)=+\infty$ otherwise. There is a close relationship between the above augmented Lagrangian function ${\tilde{L}}_{\sigma }(u,v,s;x,y)$ given by the above (45) and the one $L_{\sigma }(u,v;x,y)$ given in (9). In fact we can show that

$$L_{\sigma }(u,v;x,y)=\underset{s}{\text{inf}}\;{\tilde{L}}_{\sigma }(u,v,s;x,y)$$

and the infimum is achieved at $s=\max(-\frac{1}{n}L^{T}v+1_{m}-\frac{1}{\sigma }x,0)$. This can be done by substituting this expression of s back into (45), resulting in $L_{\sigma }(u,v;x,y)$. Using this relationship between the two augmented Lagrangian functions, next we show that the ALM using ${\tilde{L}}_{\sigma }(u,v,s;x,y)$ in (45) would be equivalent to the ALM of the main paper (7) and (8). With ${\tilde{L}}_{\sigma }(u,v,s;x,y)$ in (45), the iterative framework of the ALM is

{(uk+1,vk+1,sk+1)≈argminu,v∈RnL~σk(u,v,s;xk,yk),xk+1=xk+σk(1nLTvk+1+sk+1−1m)yk+1=yk+σk(uk+1−vk+1).}

The first step $\left(u^{k+1},v^{k+1},s^{k+1})\approx {\text{argmin}}_{u,v\in R^{n}}{\tilde{L}}_{\sigma_{k}}(u,v,s;x^{k},y^{k}\right)$ can be computed via

$$(u^{k+1},v^{k+1})\approx \underset{u,v\in R^{n}}{\text{argmin}}\;L_{\sigma_{k}}(u,v;x^{k},y^{k})\;s^{k+1}=\max\left(-\frac{1}{n}L^{T}v^{k+1}+1_{m}-\frac{1}{\sigma }x^{k},0\right).$$

By substituting the expression for $s^{k+1}$ into the update of $x^{k+1}$, we can see that

$$x^{k+1}=x^{k}+\sigma_{k}\left(\frac{1}{n}L^{T}v^{k+1}+s^{k+1}-1_{m}\right)\;\;=\max\left(\frac{\sigma_{k}}{n}L^{T}v^{k+1}-\sigma_{k}1_{m}+x^{k},0\right),$$

which is exactly the update of $x^{k+1}$ in the ALM of the main paper; see line 5 of Algorithm 1. Therefore, with either the “conventional” augmented Lagrangian function or the one in (9), the ALM is essentially the same.

## References

[R1] Akritas MG and Bershady MA. Linear regression for astronomical data with measurement errors and intrinsic scatter. The Astrophysical Journal, 470(2):706, 1996.

[R2] Andersen ED and Andersen KD. The MOSEK interior point optimizer for linear programming: an implementation of the homogeneous algorithm, pages 197–232. Springer US, Boston, MA, 2000.

[R3] Anderson L Hogg DW Leistedt B, Price-Whelan AM, and Bovy J. Improving Gaia parallax precision with a data-driven model of stars. The Astronomical Journal, 156(4): 145, 2018.

[R4] Bertsekas DP. The auction algorithm: a distributed relaxation method for the assignment problem. Annals of Operations Research, 14(1-4):105–123, 1988.

[R5] Bertsekas DP. Nonlinear Programming (3rd edition). Athena Scientific, 2016.

[R6] Böhning D. Numerical estimation of a probability measure. Journal of Statistical Planning and Inference, 11(1):57–69, 1985.

[R7] Böhning D.. The EM algorithm with gradient function update for discrete mixtures with known (fixed) number of components. Statistics and Computing, 13(3):257–265, 2003.

[R8] Bovy J, Hogg DW, and Roweis ST. Extreme deconvolution: Inferring complete distribution functions from noisy, heterogeneous and incomplete observations. Annals of Applied Statistics, 5(2B):1657–1677, 2011.

[R9] Brown AG, Vallenari A, Prusti T, De Bruijne J, Mignard F, Drimmel R, Babusiaux C, Bailer-Jones C, Bastian U, Biermann M, Gaia data release 1-summary of the astrometric, photometric, and survey properties. Astronomy & Astrophysics, 595:A2, 2016.

[R10] Brown LD and Greenshtein E. Nonparametric empirical Bayes and compound decision approaches to estimation of a high-dimensional vector of normal means. Annals of Statistics, 37(4):1685–1704, 2009.

[R11] Carlin BP and Louis TA. Bayes and Empirical Bayes Methods for Data Analysis, volume 69 of Monographs on Statistics and Applied Probability. Chapman & Hall, London, 1996.

[R12] Chares R. Cones and interior-point algorithms for structured convex optimization involving powers and exponentials. PhD thesis, Université Catholique de Louvain Louvain-la-Neuve, Belgium, 2009.

[R13] Cui Y, Ding C, and Zhao X. Quadratic growth conditions for convex matrix optimization problems associated with spectral functions. SIAM Journal on Optimization, 27(4):2332–2355, 2017.

[R14] Cui Y, Sun DF, and Toh K-C. On the R-superlinear convergence of the KKT residuals generated by the augmented Lagrangian method for convex composite conic programming. Mathematical Programming, 178(1):381–415, 2019.

[R15] Dahl J and Andersen ED. A primal-dual interior-point algorithm for nonsymmetric exponential-cone optimization. Mathematical Programming, 194(1):341–370, 2022.

[R16] Deb N, Ghosal P, and Sen B. Rates of estimation of optimal transport maps using plugin estimators via barycentric projections. Advances in Neural Information Processing Systems, 34:29736–29753, 2021.

[R17] Dedecker J and Michel B. Minimax rates of convergence for Wasserstein deconvolution with supersmooth errors in any dimension. Journal of Multivariate Analysis, 122:278–291, 2013.

[R18] Dempster AP, Laird NM, and Rubin DB. Maximum likelihood from incomplete data via the EM algorithm. Journal of the Royal Statistical Society: Series B (Methodological), 39(1):1–22, 1977.

[R19] Dvurechensky P, Ostroukhov P, Safin K, Shtern S, and Staudigl M. Self-concordant analysis of Frank-Wolfe algorithms. In International Conference on Machine Learning, volume 119, pages 2814–2824, 2020.

[R20] Efron B. Large-Scale Inference: Empirical Bayes Methods for Estimation, Testing, and Prediction, volume 1 of Institute of Mathematical Statistics (IMS) Monographs. Cambridge University Press, Cambridge, 2010.

[R21] Efron B. Bayes, oracle Bayes and empirical Bayes. Statistical Science, 34(2):177–201, 2019.

[R22] Efron B and Hastie T. Computer Age Statistical Inference—Algorithms, Evidence, and Data Science, volume 6 of Institute of Mathematical Statistics (IMS) Monographs. Cambridge University Press, Cambridge, 2021.

[R23] Facchinei F and Pang J-S. Finite-dimensional Variational Inequalities and Complementarity Problems. Springer Science & Business Media, 2007.

[R24] Hestenes MR. Multiplier and gradient methods. Journal of Optimization Theory and Applications, 4(5):303–320, 1969.

[R25] Hiriart-Urruty J-B and Lemaréchal C. Convex Analysis and Minimization Algorithms. II, volume 306. Springer-Verlag, Berlin, 1993.

[R26] Hogg DW, Myers AD, and Bovy J. Inferring the eccentricity distribution. The Astrophysical Journal, 725(2):2166, 2010.

[R27] Jiang W and Zhang C-H. General maximum likelihood empirical Bayes estimation of normal means. Annals of Statistics, 37(4):1647–1684, 2009.

[R28] Johnstone IM and Silverman BW. Needles and straw in haystacks: Empirical Bayes estimates of possibly sparse sequences. Annals of Statistics, 32(4):1594–1649, 2004.

[R29] Kelly BC. Measurement error models in astronomy. In Statistical chal lenges in modern astronomy V, pages 147–162. Springer, 2012.

[R30] Kiefer J and Wolfowitz J. Consistency of the maximum likelihood estimator in the presence of infinitely many incidental parameters. Annals of Mathematical Statistics, 27(4):887–906, 1956.

[R31] Kim Y, Carbonetto P, Stephens M, and Anitescu M. A fast algorithm for maximum likelihood estimation of mixture proportions using sequential quadratic programming. Journal of Computational and Graphical Statistics, 29(2):261–273, 2020. 33762803 10.1080/10618600.2019.1689985 PMC7986967

[R32] Koenker R and Gu J. REBayes: An R package for empirical Bayes mixture methods. Journal of Statistical Software, 82(8):1–26, 2017.

[R33] Koenker R and Mizera I. Convex optimization, shape constraints, compound decisions, and empirical Bayes rules. Journal of the American Statistical Association, 109(506): 674–685, 2014.

[R34] Kojima M and Shindo S. Extension of Newton and quasi-Newton methods to systems of PC^1^ equations. Journal of the Operations Research Society of Japan, 29(4):352–375, 1986.

[R35] Kummer B.. Newton’s method for non-differentiable functions. Advances in Mathematical Optimization, 45(1988):114–125, 1988.

[R36] Laird N.. Nonparametric maximum likelihood estimation of a mixing distribution. Journal of the American Statistical Association, 73(364):805–811, 1978.

[R37] Lashkari D and Golland P. Convex clustering with exemplar-based models. In Advances in Neural Information Processing Systems, volume 20, pages 825–832, 2007. PMC4485428 26140013

[R38] Lesperance ML and Kalbfleisch JD. An algorithm for computing the nonparametric MLE of a mixing distribution. Journal of the American Statistical Association, 87(417): 120–126, 1992.

[R39] Li X, Sun DF, and Toh K-C. On efficiently solving the subproblems of a level-set method for fused lasso problems. SIAM Journal on Optimization, 28(2):1842–1862, 2018a.

[R40] Li X, Sun DF, and Toh K-C. A highly efficient semismooth Newton augmented Lagrangian method for solving lasso problems. SIAM Journal on Optimization, 28(1): 433–458, 2018b.

[R41] Li X, Sun DF, and Toh K-C. QSDPNAL: A two-phase augmented Lagrangian method for convex quadratic semidefinite programming. Mathematical Programming Computation, 10:703–743, 2018c.

[R42] Li X, Sun DF, and Toh K-C. An asymptotically superlinearly convergent semismooth Newton augmented lagrangian method for linear programming. SIAM Journal on Optimization, 30(3):2410–2440, 2020.

[R43] Lindsay BG. The geometry of mixture likelihoods: a general theory. Annals of Statistics, 11(1):86–94, 1983.

[R44] Lindsay BG. Mixture Models: Theory, Geometry and Applications, volume 5. Institute of Mathematical Statistics, 1995.

[R45] Liu C and Rubin DB. The ECME algorithm: a simple extension of EM and ECM with faster monotone convergence. Biometrika, 81(4):633–648, 1994.

[R46] Liu L and Rubin DB. Maximum likelihood estimation of factor analysis using the ECME algorithm with complete and incomplete data. Statistica Sinica, pages 729–747, 1998.

[R47] Liu L and Zhu Y. Partially projected gradient algorithms for computing nonparametric maximum likelihood estimates of mixing distributions. Journal of Statistical Planning and Inference, 137(7):2509–2522, 2007.

[R48] Majewski SR, Schiavon RP, Frinchaboy PM, Prieto CA, Barkhouser R, Bizyaev D, Blank B, Brunner S, Burton A, Carrera R, The apache point observatory galactic evolution experiment (APOGEE). The Astronomical Journal, 154(3):94, 2017.

[R49] Manole T, Balakrishnan S, Niles-Weed J, and Wasserman L. Plugin estimation of smooth optimal transport maps. arXiv preprint arXiv:2107.12364, 2021.

[R50] McCann RJ and Guillen N. Five lectures on optimal transportation: geometry, regularity and applications. Analysis and geometry of metric measure spaces: lecture notes of the séminaire de Mathématiques Supérieure (SMS) Montréal, pages 145–180, 2011.

[R51] Munkres J.. Algorithms for the assignment and transportation problems. Journal of the Society for Industrial and Applied Mathematics, 5:32–38, 1957.

[R52] Nocedal J and Wright SJ. Numerical Optimization. Springer, 2006.

[R53] Peyré G and Cuturi M. Computational Optimal Transport: With Applications to Data Science. Foundations and Trends in Machine Learning, 11(5-6):355–607, 2019.

[R54] Polyanskiy Y and Wu Y. Self-regularizing property of nonparametric maximum likelihood estimator in mixture models. arXiv preprint arXiv:2008.08244, 2020.

[R55] Powell MJ. A method for nonlinear constraints in minimization problems. Optimization, pages 283–298, 1969.

[R56] Qi L and Sun J. A nonsmooth version of Newton’s method. Mathematical Programming, 58(1):353–367, 1993.

[R57] Ratcliffe BL, Ness MK, Johnston KV, and Sen B. Tracing the assembly of the milky way’s disk through abundance clustering. The Astrophysical Journal, 900(2):165, 2020.

[R58] Redner RA and Walker HF. Mixture densities, maximum likelihood and the EM algorithm. SIAM Review, 26(2):195–239, 1984.

[R59] Robbins H.. A generalization of the method of maximum likelihood-estimating a mixing distribution. In Annals of Mathematical Statistics, volume 21, pages 314–315, 1950.

[R60] Rockafellar RT. Augmented Lagrangians and applications of the proximal point algorithm in convex programming. Mathematics of Operations Research, 1(2):97–116, 1976.

[R61] Rockafellar RT and Wets RJ-B. Variational Analysis, volume 317. Springer Science & Business Media, 2009.

[R62] Royset JO and Wets RJ-B. An Optimization Primer. Springer, 2022.

[R63] Santambrogio F.. Optimal Transport for Applied Mathematicians. Birkäuser, NY, 2015.

[R64] Sarkar A, Pati D, Chakraborty A, Mallick BK, and Carroll RJ. Bayesian semiparametric multivariate density deconvolution. Journal of the American Statistical Association, 113(521):401–416, 2018. 30078920 10.1080/01621459.2016.1260467 PMC6075844

[R65] Soloff JA, Guntuboyina A, and Sen B. Multivariate, heteroscedastic empirical Bayes via nonparametric maximum likelihood. arXiv preprint arXiv:2109.03466, 2021.

[R66] Stephens M.. False discovery rates: a new deal. Biostatistics, 18(2):275–294, 2017. 27756721 10.1093/biostatistics/kxw041 PMC5379932

[R67] Tran-Dinh Q, Kyrillidis A, and Cevher V. Composite self-concordant minimization. Journal of Machine Learning Research, 16(1):371–416, 2015.

[R68] Varadhan R and Roland C. Simple and globally convergent methods for accelerating the convergence of any EM algorithm. Scandinavian Journal of Statistics. Theory and Applications, 35(2):335–353, 2008.

[R69] Villani C.. Topics in Optimal Transportation. American Mathematical Society, 2003.

[R70] Villani C.. Optimal Transport: Old and New. Springer, 2009.

[R71] Wang H, Ibrahim S, and Mazumder R. Nonparametric finite mixture models with possible shape constraints: A cubic Newton approach. arXiv preprint arXiv:2107.08535, 2021.

[R72] Wang Y.. On fast computation of the non-parametric maximum likelihood estimate of a mixing distribution. Journal of the Royal Statistical Society: Series B (Statistical Methodology), 69(2):185–198, 2007.

[R73] Yuan G.. A homogeneous interior-point method for conic programming involving exponential cone constraints. Master’s thesis, National University of Singapore, 2017.

[R74] Zhang N, Zhang Y, Sun DF, and Toh K-C. An efficient linearly convergent regularized proximal point algorithm for fused multiple graphical lasso problems. SIAM Journal on Mathematics of Data Science, 3(2):524–543, 2021.

[R75] Zhang Y, Zhang N, Sun DF, and Toh K-C. An efficient Hessian based algorithm for solving large-scale sparse group Lasso problems. Mathematical Programming, 179(1): 223–263, 2020.

[R76] Zhang Y, Cui Y, Sen B, and Toh K-C. On efficient and scalable computation of the nonparametric maximum likelihood estimator in mixture models. arXiv preprint arXiv:2208.07514, 2022. PMC12975118 41815789

